# Chromosome‐level genome assembly of the photobiont microalga *Trebouxia* sp. ‘A48’ from the lichen *Xanthoria parietina*

**DOI:** 10.1111/nph.70728

**Published:** 2025-11-10

**Authors:** Gulnara Tagirdzhanova, Jasper Raistrick, Nicholas J. Talbot

**Affiliations:** ^1^ The Sainsbury Laboratory, University of East Anglia Norwich Research Park Colney Lane Norwich NR4 7UH UK; ^2^ Department of Ecology, Environment and Plant Sciences Stockholm University Stockholm 106 91 Sweden

**Keywords:** algae, chlorophyte, genomics, lichen, long‐read sequencing, mutualism, photobiont, symbiosis

## Abstract

- Lichens are symbiotic assemblies consisting of multiple organisms, chiefly a fungus and a photosynthetic microorganism, or photobiont. Among diverse photobionts, the most prevalent is the chlorophyte alga *Trebouxia*.
- We produced a chromosome‐level assembly of *Trebouxia* sp. ‘A48’, a photobiont of *Xanthoria parietina*. The genome was assembled into 20 contigs, of which 16 had telomeric repeats at both ends and likely represent complete chromosomes. We compared this genome with those of other *Trebouxia* species and analyzed it to investigate adaptations to the lichen lifestyle. We then used the genome to profile gene expression in axenic culture and in lichen thalli.
- The predicted secretome is enriched in hydrolases and redox enzymes and contains carbohydrate‐binding proteins potentially involved in cell‐to‐cell recognition and adhesion. We identified genes potentially involved in carbon concentrating and confirmed two instances of ancient horizontal gene transfer from fungi.
- The genome and the strain of *Trebouxia* sp. ‘A48’ provide a resource for the community to research algal evolution and lichen symbiosis.

## Introduction

The textbook example of symbiosis, the lichen symbiosis, is centered around the relationship between a fungal partner called the mycobiont and a photosynthetic symbiont, known as the photobiont (Spribille *et al*., 2022). Lichen photobionts are diverse and include both prokaryotic cyanobacteria and eukaryotic microalgae, and among both groups the lichen lifestyle has emerged several times independently (Scharnagl *et al*., 2023). Our understanding of the mechanics and evolution of the lichen symbiosis relies on generating sufficient information from both key symbionts. However, compared with their fungal partners, lichen photobionts have received little attention. This gap has begun to close recently with new studies investigating the genomics and evolution of chlorophyte photobionts (Gazquez *et al*., 2024; Poquita‐Du *et al*., 2024; Puginier *et al*., 2024).

High‐quality genomic data enables research on molecular mechanisms involved in lichen symbioses, which are otherwise challenging to study because our ability to manipulate lichen symbionts experimentally is severely limited. Transcriptomics has, for example, allowed profiling lichen algae responses to desiccation (Carniel *et al*., 2016; Del Campo *et al*., 2023), UV exposure (Leksin *et al*., 2024), and temperature shifts (Armaleo *et al*., 2019; Kono *et al*., 2020; Chavarria‐Pizarro *et al*., 2022; Almer *et al*., 2023). High‐quality reference genomes allow for more robust inference from transcriptomic and proteomic data.

The Chlorophyta alga *Trebouxia* (Order Trebouxiales, Class Trebouxiophyceae) is the most common lichen photobiont, estimated to participate in nearly half of all described lichen symbioses (Sanders & Masumoto, 2021). *Trebouxia* algae are diverse, and while only *c*. 35 species have been formally described, the genus also includes 110 candidate species split into five clades: clade A (*T. arboricola/gigantea* group), clade C (*T. corticola/galapagensis/usneae* group), clade I (*T. impressa/gelatinosa* group), clade S (*T. implex/letharii/jamesii* group), and clade D (*T. delisei* group) (Muggia *et al*., 2020; Xu *et al*., 2020; Barreno *et al*., 2022; Garrido‐Benavent *et al*., 2022; Chiva *et al*., 2025; Pazos *et al*., 2025). Individual lineages within the genus can often enter the symbiosis with different mycobionts. Conversely, a single mycobiont might associate with one or several lineages of *Trebouxia* (Sanders & Masumoto, 2021). The mechanisms determining which photobiont–mycobiont associations can occur remain largely unknown. Whether and to what extent *Trebouxia* has an aposymbiotic phase is also debated (Sanders & Masumoto, 2021). The basis of the lichen symbiosis is assumed to be nutritional: the product of the photobiont's photosynthesis (in the case of *Trebouxia*, ribitol) is accumulated by the mycobiont and used either for respiration or for surviving desiccation (Richardson & Smith, 1968; Spribille *et al*., 2022). The photobiont genes involved in the synthesis and release of ribitol have not yet been conclusively identified. *Trebouxia* is known to be mixotrophic (Fox, 1967), leading to the hypothesis that a flow of carbohydrates in the opposite direction, from mycobiont to the photobiont, may also occur (Ahmadjian, 2002). *Trebouxia* can reproduce sexually in culture (Boccato *et al*., 2025), but to what extent sexual reproduction occurs in nature is largely unknown.

In spite of the importance of *Trebouxia* as a photobiont and the great diversity of this genus, our understanding of its biology has been limited, as only a handful of high‐quality genome sequences are available. The majority of genomic assemblies available at NCBI originate from shotgun metagenomes and are therefore highly fragmented. Available resources also include a shotgun genome assembly from a culture of *Trebouxia* sp. TZW2008 (clade C) (Kono *et al*., 2017), as well as long‐read genomes of six strains, three from Clade S (S09, S19, S12) and three from Clade A (A06, A10, A04) (Poquita‐Du *et al*., 2024). *Trebouxia lynnae* (clade A) has three genomes available, only one of which is published (Gazquez *et al*., 2024). Most recently, a chromosome‐level assembly of *Trebouxia* was generated as part of the Aquatic Symbiosis Genomics project (Table 1). This assembly was extracted from a long‐read metagenome of *Xanthoria parietina* using binning and HiFi data (McKenna *et al*., 2024) and deposited in NCBI as *T*. sp. SL0000003.

**Table 1:** Characteristics of the genomic assembly of *Trebouxia* sp. ‘A48’ compared with *Trebouxia* sp. SL0000003.

Characteristic	*Trebouxia* sp. ‘A48’	*Trebouxia* sp. SL0000003
Genome size, bp	69 092 103	63 705 246
Contig number in nuclear genome	20	20
Contigs with telomeric repeats on both ends	16	3
N50, Mbp	3.71	1.19
BUSCO completeness	97.2%	92.8%
BUSCO duplication	0.6%	0.7%
Repeat content	12.72%	10.6%
GC (guanine‐cytosine) content	49.68%	50.3%
Number of genes	12 820	10 061
Number of proteins	14 492	9928
Source	This study	Aquatic Symbiosis Genomic Project
Source type	Isolate	Metagenome
Accession Number	PRJEB95800	PRJEB86095

Bp, base pairs.

**Fig. 1:**
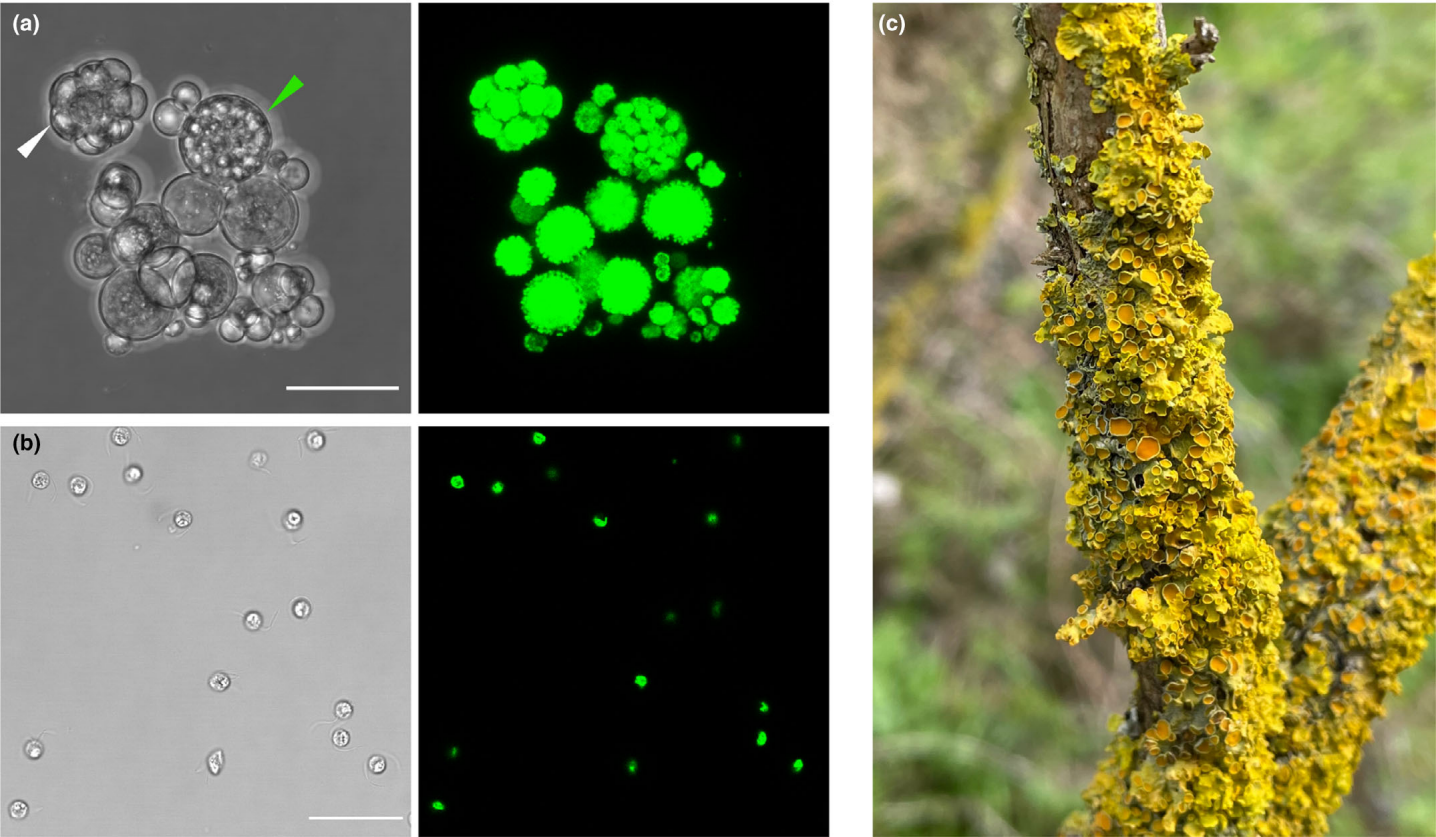
*Trebouxia* sp. ‘A48’. (a, b) Micrographs of *Trebouxia* sp. ‘A48’ cultured from an *Xanthoria parietina* thallus. The left panel shows bright‐field microscopy; the right panel shows Chl autofluorescence. Bar, 25 μm. (a) A cluster containing mature and young *Trebouxia* sp. ‘A48’ cells, as well as an autosporangium (white arrow) and a zoosporangium (green arrow). (b) Flagellated cells of *Trebouxia* sp. ‘A48’. (c) A thallus of *X. parietina*.

In this study, we present the genome sequence of a *Trebouxia* photobiont of the common sunburst lichen *X. parietina* (Fig. 1), which has long served as a model system for studies of lichen development, physiology, and genetics (Korhonen & Kallio, 1987; Honegger, 1996; Honegger *et al*., 2004). *X. parietina* lichens involve several *Trebouxia* lineages, with the majority coming from the *T. decolorans* species complex (Nyati *et al*., 2014). We have generated a chromosome‐level assembly of the photobiont genome and used this to investigate the life cycle, symbiotic lifestyle, and evolutionary history of the symbiotic alga.

## Materials and Methods

### Strain isolation

The algal strain was isolated from a thallus of *Xanthoria parietina* (L.) Th. Fr. lichen collected from an oak branch in Norwich Research Park (Norwich, UK; 52.622991°N, 1.221588°E). We followed the isolation procedure of Yoshimura *et al*. (2002): a thallus fragment was washed under a jet of water, homogenized, and plated on solid Bold's Mineral Medium. The plates were incubated on a benchtop for 1 wk and then transferred to a growth chamber, where they were incubated at 18°C with a 12 h : 12 h, light : dark cycle at 550 μmol m^−2^ s^−1^ light level. After 6 wk of incubation, several algal colonies were re‐plated on solid *Trebouxia* Organic Nutrient Medium (TONM) (Yoshimura *et al*., 2002) with rifampicin (the final concentration of 50 μg ml^−1^) and carbendazim (40 μg ml^−1^). A single colony from one of the contamination‐free plates was then selected and re‐plated. To bulk the culture, we used liquid TONM medium with rifampicin (50 μg ml^−1^) and carbendazim (25 μg ml^−1^), incubated in the conditions described above. To confirm the axenic status of the culture, we cultured it on solid lysogeny broth (LB) medium and incubated it for 2 d at 28°C and 37°C. The algal strain was deposited at The Culture Collection of Algae at the University of Göttingen, Germany (SAG) as SAG 2671.

### Nucleic acid extraction and sequencing

For DNA extraction, we used a liquid algal culture which had been maintained in liquid TONM medium for 25 d. We concentrated 100 ml of liquid algal culture by centrifugation at 3739 *g* for 5 min. The cell pellet was snap‐frozen and freeze‐dried. We homogenized *c*. 80 mg of dried material using a Geno/grinder at 1300 rpm for 1 min. Genomic DNA was extracted using a NucleoBond High Molecular Weight DNA Kit and purified using a Qiagen genomic Tip20 kit and a Circulomics Short read eliminator kit with a 25‐kb cut‐off. A sequencing library was prepared with a Native Barcoding Kit 24 V14 and sequenced on a PromethION P2 Solo (P2S) device with a PromethION Flow Cell FLO‐PRO114M to 22.4 Gbp of raw data. Base calling was performed with Dorado v.7.4.13. Genomic sequencing and assembly were carried out by Future Genomics (Leiden, the Netherlands).

In total, we produced four transcriptomic libraries. For one, we harvested and homogenized algal material as described above. This library was used for improving genome annotation. The three libraries that were used for the differential gene expression analysis were produced from algal cultures grown on solid TONM medium for 2 months. Total RNA was extracted with an RNEasy Plant Mini Kit; the mRNA library was prepared and sequenced by Novogene on an Illumina machine to 8 Gbp of PE150 data.

### Genome assembly

Draft assembly was performed with Hifiasm v.0.24.0 (Cheng *et al*., 2021). We used samtools v.1.12 (Li *et al*., 2009), Minimap2 v.2.24–41122 (Li, 2018), MetaBAT v.2.15 (Kang *et al*., 2019), and blast+ v.2.9.0 (Camacho *et al*., 2009) to examine the contigs, including their coverage, GC content, and BLAST matches to the NCBI nt database (Supporting Information Fig. S1a). For the nuclear genome, we retained contigs with GC content 48–50% and coverage 180–210×. An additional contig with 390× coverage was identified as a hybrid, where a fragment of the nuclear and plastid genome was fused. By examining the read mapping file, we identified the misassembly point (Fig. S1b) and manually split the contig. To identify other cases of misassembly resulting from telomeric repeats, we identified and examined every instance where telomeric repeats were present more than 500 bp away from the contig terminus. Several similar misassembly points were thus identified and removed (Fig. S1c). Notably, one of the contigs included in the final genome assembly had a majority of BLASTn hits to animal genomes (Fig. S1a). We considered these hits erroneous and included the contig in the assembly for the following reasons: (1) the contig is over 2 Mbp long with no plausible misassembly sites and has the same coverage depth as the rest of the nuclear genome; (2) all but one hit to animal genomes originate from a fragment only 1300 bp long; (3) all these hits were to fish genomes, which can plausibly be contaminated with algal sequences; (4) high‐quality hits to lichen‐associated algae can be obtained from the same region. We confirmed the completeness of the nuclear genome assembly using the marker gene‐based BUSCO v.4.0.6 algorithm (Seppey *et al*., 2019) with the chlorophyta_odb10 database. To align the nuclear genome assembly to the genome of *T*. sp. SL0000003 (PRJEB86095), we used Minimap2 v.2.24–41122 (Li, 2018) with the ‐x asm20 flag. The synteny plot was generated with syntenyPlotteR (Quigley *et al*., 2023).

As the mitochondrial genome failed to assemble as a single contig, we reassembled it from reads identified as mitochondrial. First, we extracted all reads that mapped to the group of contigs with BLAST matches to Chlorophyta mitochondria (Fig. S1a). We then selected the top 30 000 reads and reassembled them using Hifiasm v.0.18.5 (Cheng *et al*., 2021). A complete copy of the chloroplast genome was recovered in the initial assembly on a circular contig.

### Genome annotation

We masked repeats using RepeatMasker v.4.0.9 (https://www.repeatmasker.org/) with a custom repeat library prepared with RepeatModeler v.2.0.3 (Flynn *et al.* 2020) from the genomic assembly. The repeat‐masked genome was then used for gene prediction using the funannotate pipeline v.1.8.15 (Palmer & Stajich, 2020). First, we used the funannotate ‘train’ module to train Augustus prediction using transcriptomic data, which was assembled within funannotate. Next, we ran gene prediction using Genemark‐ES v.4.62 (Lomsadze *et al*., 2005) and the funannotate ‘predict’ module, which collected predictions from Augustus v.3.3.2 (Stanke & Waack, 2003), GlimmerHMM v.3.0.4 (Majoros *et al*., 2004), and SNAP 2006‐07‐28 (Korf, 2004). We created consensus models with EVidence Modeler v.1.1.1 (Haas *et al*., 2008) and annotated tRNA with tRNAscan‐SE v.2.0.9 (Chan & Lowe, 2019). The predictions were refined using transcriptomic data with the funannotate ‘update’ module. For functional annotations, we used InterProScan v.5.52–86.0 (Paysan‐Lafosse *et al*., 2023) and the funannotate ‘annotate’ module, which collected predictions from PFAM v.35.0 (Mistry *et al*., 2021), UniProtDB v2023_01 (UniProt Consortium, 2023), MEROPS v.12.0 (Rawlings *et al*., 2014), dbCAN v.11.0 (Yin *et al*., 2012), and BUSCO chlorophyta_odb10 (Seppey *et al*., 2019). We also annotated KEGG ontology using KAAS webserver (Moriya *et al*., 2007). To detect telomeric repeats, we used the script from Hiltunen *et al*. (2021), with ‘CCCTAAA’ as a query. *Trebouxia* sp. SL0000003 genome was annotated using the same protocol, with two modifications: due to the lack of RNAseq data, we skipped the ‘train’ and ‘update’ modules; instead, we supplied the predicted proteome of *T*. sp. ‘A48’ as evidence for the ‘predict’ module. To identify genes involved in meiosis and syngamy, we relied on the InterProScan annotations or, in the case of *HOP1*, *HOP2*, and *MER3*, we used genes from *Coccomyxa subellipsoidea* (Fučíková *et al*., 2015) as BLAST queries. To search for gamete‐specific genes *FUS1* and *MTD1*, we used sequences from *Chlamydomonas reinhardtii* (AAL14635.1 and AAC49416.1). We searched both the predicted proteome and the original pre‐decontamination assembly.

To characterize ploidy, we followed the procedure of Ament‐Velásquez *et al*. (2021). We aligned the long read data on the nuclear genome assembly using Minimap2 v.2.24–41 122 (Li, 2018) and removed duplicated reads with Picard v.2.21.2 (https://broadinstitute.github.io/picard/). We called variants using VarScan v.2.3.9 (Koboldt *et al*., 2012) with the flags – *P*‐value 0.1 – min‐var‐freq 0.005. The results were processed using the vcfR library v.1.15.0 (Knaus & Grünwald, 2017); only positions with coverage depth 200–240× were considered.

For secretome prediction, we used WolfPSORT (Horton *et al*., 2007), deepTMHMM (Hallgren *et al*., 2022), and SignalP v.5 (Almagro Armenteros *et al*., 2019). We considered a protein as secreted if it has a signal peptide identified by SignalP, no transmembrane domains identified by deepTMHMM, and the highest probability of being secreted according to WolfPSORT. We used ClusterProfiler v.4.2.2 (Yu *et al*., 2012) for enrichment analysis. To annotate conserved domains in the uncharacterized secreted proteins and to confirm that full‐length domains were present in secreted CAZymes, we used NCBI Batch search against the Conserved Domain Database (Wang *et al*., 2023).

We annotated the plastid and mitochondrial genomes using MFannot (Lang *et al*., 2023) and GeSeq (Tillich *et al*., 2017). To cross‐reference the annotations with the transcriptome data, we aligned RNA data onto the organelle genomes using star v.2.5.4b (Dobin *et al*., 2013). The final annotations were curated manually and visualized with OGDRAW (Greiner *et al*., 2019).

### Phylogenetic analysis

To provide a taxonomic assignment for the genome, we assembled a reference set of *Trebouxia* internal transcribed spacer (ITS) sequences following Muggia *et al*. (2020). For each of the operational taxonomic units (OTUs), we selected at most five representatives for which an ITS sequence was available and added *Myrmecia* sp. 1 IM‐2024 (PQ154957.1) as an outgroup (Table S1). To identify ITS sequences within our genome assembly and *T*. sp. SL0000003, we used a BLASTn search with *T*. sp. P‐121‐IIcd ITS (AJ969550.1) as a query. We aligned the sequences using MAFFT v.7.271 (Katoh & Standley, 2013) and trimmed the alignment to remove positions absent in over 20% of sequences using trimAl v.1.2 (Capella‐Gutiérrez *et al*., 2009). The maximum‐likelihood phylogenetic tree was constructed with IQ‐TREE v.2.2.2.2 (Minh *et al*., 2020) using 10 000 rapid bootstraps. To construct a phylogenomic tree and carry out orthogroups analysis, we combined 24 reference genomes from the literature (Table S2) and the predicted proteomes from our genome assembly and *T*. sp. SL0000003, and analyzed them with OrthoFinder v.2.5.4 (Emms & Kelly, 2019). As reference genomes, we included a representative set of algal genomes across Chlorophyta, an outgroup from Charophyta, and every species of *Trebouxia* for which we had genome annotations. For *T. lynnae*, which lacked publicly available genome annotations, we used BUSCO genes identified using BUSCO v.4.0.6 (Seppey *et al*., 2019) with the chlorophyta_odb10 database. Phylogenies were visualized using iTOL v.7 (Letunic & Bork, 2024).

### Horizontal gene transfer (HGT) analysis

To identify the HGT candidates reported by Beck *et al*. (2015) in the newly produced genome, we used their nucleotide sequences (KF573967, KF573968, KF573969) as a BLAST query against the predicted transcriptome and the complete genomic assembly. The identified proteins were searched against NCBI nonredundant protein (nr) and nucleotide (nt) databases (access date 2025/04/15). We ran three searches: (1) including all taxa; (2) excluding fungi; (3) including only Viridiplantae hits. We collected the top 100 hits from all searches and excluded all hits with query coverage below 75%. The remaining protein sequences were combined with sequences from *T*. sp. SL0000003 and *T*. sp. ‘A48’ proteomes. We aligned the sequences and reconstructed the phylogeny as described above.

### Transcriptomic analysis

The transcriptomic libraries produced from algal culture grown on solid medium were trimmed with cutadapt v.1.17 (Martin, 2011) to remove adaptor contamination. To remove rRNA contamination, we first extracted rRNA operons from the nuclear and organelle genomes by using BLASTn search with published *Trebouxia* rRNA sequences (NCBI accessions: EU725860.1, MK320925.1, KX147269.1, EU123942.1, Z95383.1, Z21553.1, JN847659.1) as queries. From the resulting file, we created a custom database and filtered the libraries to remove matching sequences using SortMeRNA v.3.0.3 (Kopylova *et al*., 2012). We complemented the transcriptomic data from pure culture with metatranscriptomic data produced from *X. parietina* thalli from a previous study (Tagirdzhanova *et al*., 2025). Briefly, metatranscriptomic libraries were produced from different parts of lichen thalli and aligned to a catalogue of metagenome‐assembled genomes. For the present analysis, we selected eight libraries (Table S3) and extracted all reads mapped to algal genomes included in the original analysis using samtools v.1.12 (Li *et al*., 2009). Each transcriptome library was pseudoaligned to the predicted transcriptome using kallisto v.0.46.2 (Bray *et al*., 2016). We identified differentially expressed genes (DEGs) using sleuth v.0.30.1 (Pimentel *et al*., 2017) with two thresholds: *P*_adj_ < 0.05 and |*b*‐value| > 1. To identify DEGs between different parts of lichen thalli, we controlled for the thallus identity following the official sleuth tutorial (https://pachterlab.github.io/sleuth_walkthroughs/pval_agg/analysis.html). To identify overrepresented functions in the lists of DEGs, we used ClusterProfiler v.4.14.6 (Yu *et al*., 2012).

### Microscopy

To visualize the *Trebouxia* strain, we used a Leica SP8 confocal microscope. Chl autofluorescence was excited at 514 nm and detected at 675–750 nm. Images were processed in ImageJ (Schneider *et al*., 2012).

## Results

### The genome sequence of *Trebouxia* sp. ‘A48’

**Fig. 2:**
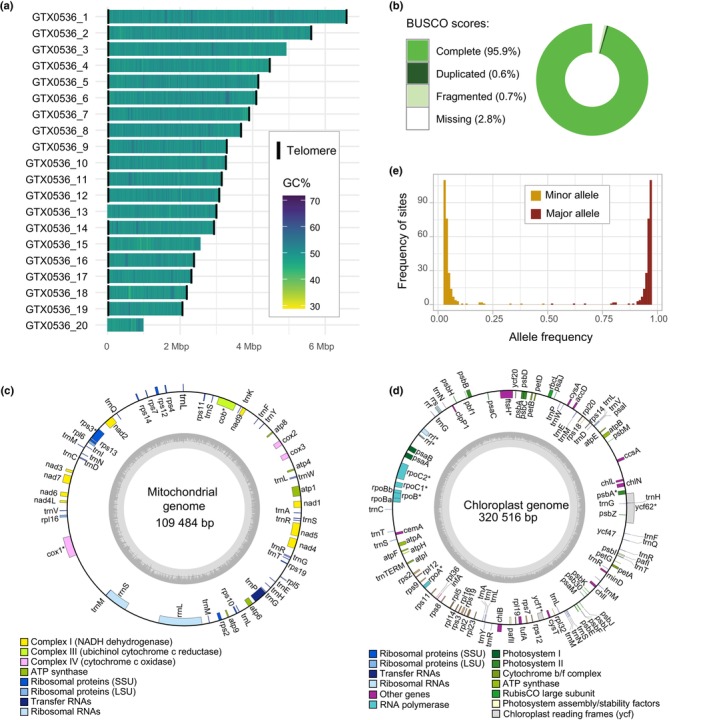
The genome of *Trebouxia* sp. ‘A48’. (a) Plot showing the number, length, and GC‐content of contigs comprising the nuclear genome. Black bars represent telomeric repeats. (b) BUSCO quality scores (chlorophyta_odb10 database). The segments of the doughnut chart represent the status of BUSCO genes: complete (green), duplicated (dark‐green), fragmented (light‐green), and missing (white). (c) Mitochondrial genome. The inner circle shows the GC‐content. The genes are colored according to their function; the genes shown on the inner side of the circle are transcribed clockwise; the genes on the outside are transcribed counterclockwise. (d) Chloroplast genome. (e) Allele frequency distribution in the nuclear genome. Yellow bars represent minor allele frequency; brown bars represent major allele frequency. We removed positions within repeat elements and those outside of the coverage depth window 200–240×. The graph only shows allelic distributions between 0.03 and 0.97.

Here we present a chromosome‐level assembly of a *Trebouxia* alga (Fig. 1a,b), which was isolated from the lichen thallus of *X. parietina* (Fig. 1c). The nuclear genome consisting of 69.1 Mbp was recovered in 20 contigs, of which 16 have telomeric repeats at both ends and likely represent complete chromosomes (Fig. 2a, Table 1). The genome is 97.2% complete according to BUSCO and has a duplication rate of 0.6% (Fig. 2b). Both plastid and mitochondrial genomes were recovered as single circular contigs, 320.5 and 109.5 kbp, respectively (Fig. 2c,d). Repeats accounted for 12.72% of the genome and included chiefly LINE elements (2.75%), simple repeats (1.82%), LTR elements (1.42%), and unclassified repeats (5.64%). *De novo* annotation of the nuclear genome produced 12 820 gene models. The genome size, repeat content, and the number of gene models are consistent with recently published genome sequences of *Trebouxia* (Poquita‐Du *et al*., 2024).

**Fig. 3:**
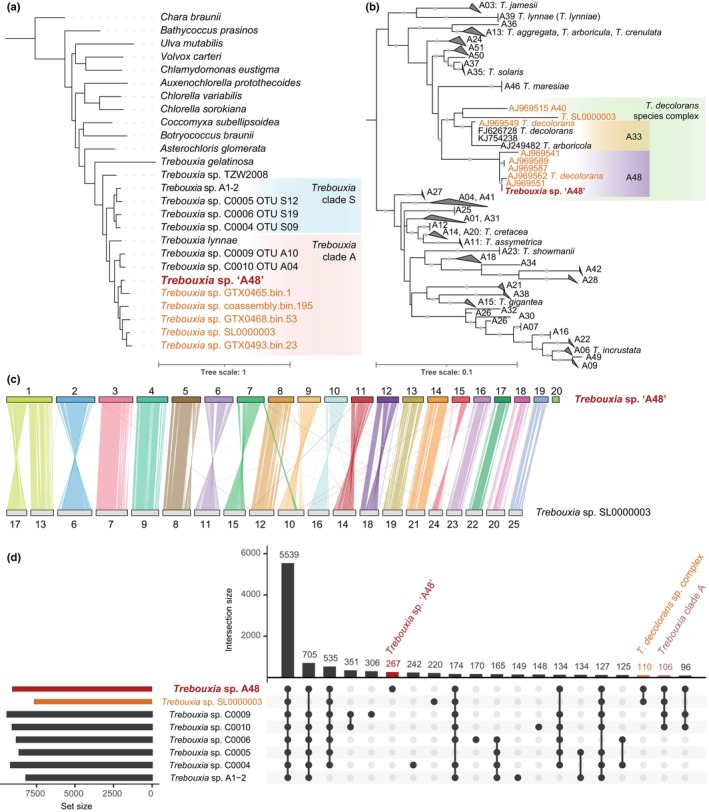
Evolutionary context of *Trebouxia* sp. ‘A48’. (a) Species tree generated with Orthofinder using the STAG algorithm based on 328 universal orthogroups. *Trebouxia* sp. ‘A48’ is shown in red; the metagenome‐assembled genomes of *Trebouxia* from *Xanthoria parietina* lichens are shown in orange. The genomes from the literature are listed in Supporting Information Table S2; the tree in Newick format can be found on GitHub (https://github.com/metalichen/2025‐Trebouxia‐genome). (b) Internal transcribed spacer (ITS)‐based maximum‐likelihood phylogeny of *Trebouxia*. Only a portion of the tree is shown; the entire tree in Newick format can be found on GitHub (https://github.com/metalichen/2025‐Trebouxia‐genome). The sequences used in the analysis are listed in Table S1. *T*. sp. ‘A48’ is shown in red; other *Trebouxia* from *Xanthoria* species are shown in orange (only shown for the *T. decolorans* clade). The clades are labeled with the operational taxonomic unit (OTU) names as defined by Muggia *et al*. (2020). Dots represent bootstrap support > 95%. (c) Synteny between the genomes of *T*. sp. ‘A48’ and the metagenome‐assembled genome of *T*. sp. SL0000003 isolated from an *X. parietina* metagenome. (d) Upset plot showing the intersection of orthogroups between *Trebouxia* species. We excluded from this graph the metagenome‐assembled genomes from Tagirdzhanova *et al*. (2025), as they had lower completeness, and *T*. sp. TZW2008, *T. lynnae*, and *T. gelatinosa*, for which we had only BUSCO annotations. Only the top 20 sets are shown.

The alga was identified as belonging to the OTU *T*. sp. ‘A48’ from the *T. decolorans* species complex, which belongs to clade ‘A’ within the *Trebouxia* genus (Muggia *et al*., 2020) (Fig. 3a,b). While *T*. sp. ‘A48’ is not yet formally described, this OTU will soon be proposed as *T. tabarcae* (P. Moya, I. Garrido‐Benavent, pers. comm.). Our isolate is closely related to photobionts of *X*. lichens collected from various geographic locations; its sister samples originated from Australia (AJ969551) and France (AJ969562) (Nyati *et al*., 2014).

### *Trebouxia* sp. ‘A48’ in the context of its genus

We compared the *T*. sp. ‘A48’ genome to the only other chromosome‐level assembly of *Trebouxia*, *T*. sp. SL0000003, which originated from a long‐read metagenome of *X. parietina* (Table 1). Our phylogenetic analysis recovered *T*. sp. SL0000003 as another member of the *T. decolorans* species complex and a sister to ‘A40’ and ‘A33’ OTUs (Fig. 3b). The two assemblies exhibit significant synteny. Each contig from the *T*. sp. ‘A48’ assembly, for example, corresponded to one contig in *T*. sp. SL0000003, with only two exceptions (Fig. 3c). First, the *T*. sp. ‘A48’ contig #1 was ‘split’ between two *T*. sp. SL0000003 contigs (OZ234913 and OZ234917), but whether this resulted from different genomic architectures or from fragmentation of the latter assembly is not clear. Second, the *T*. sp. ‘A48’ contig #20 did not have a match in the *T*. sp. SL0000003 genome, which might explain the lower completeness score received by *T*. sp. SL0000003. In addition, structural changes were present in contigs #5 and #12. The *T*. sp. ‘A48’ genome is larger, has a higher repeat content, and more genes (Table 1).

The two genomes shared 7216 orthogroups, with 1864 families unique to *T*. sp. ‘A48’ and 449 unique to *T*. sp. SL0000003. However, the absence of some gene families from *T*. sp. SL0000003 might have resulted from the lower assembly completeness. By comparing *T*. sp. ‘A48’ to other *Trebouxia* species, we identified 267 gene families unique to this isolate (Fig. 3d). In total, 110 orthogroups were present in both *T*. sp. ‘A48’ and *T*. sp. SL0000003 and no other genomes (Table S4). These included three orthogroups (with seven *T*. sp. ‘A48’ genes) of various protease‐encoding genes, one orthogroup containing three putative transporters with unknown functions, one predicted concanavalin A‐like lectin, and one putative chitinase from the family GH19 with a LysM domain, which is involved in binding peptidoglycans and chitin.

### Genomic evidence for life cycle characteristics of *Trebouxia*

Allelic frequency distribution in the genome indicates that *T*. sp A48 is haploid (Fig. 2e). While this result is consistent with the assumption frequently made regarding lichen algae (Fernández‐Mendoza *et al*., 2011; Dal Grande *et al*., 2013; Greshake Tzovaras *et al*., 2020), it stands in contrast to recent reports that *T. lynnae*, another member of clade ‘A', has a diplontic life cycle (Gazquez *et al*., 2024). Similar to *T. lynnae*, the genome of *T*. sp. ‘A48’ encodes meiosis and mating‐associated genes, including the nuclear fusion gene *GEX1*, gamete fusion gene *HAP2*, meiotic recombination protein *DMC1*, meiotic nuclear division gene *MND1*, and the meiosis‐specific protein *ZIP4*, but not *HOP1*, which was previously reported to be missing from another Trebouxiales genome (Fučíková *et al*., 2015) (Tables S5, S6). In addition, *T*. sp. ‘A48’ genome encoded the meiosis‐specific gene *HOP2*, which was lacking in *T. lynnae*. Across the genome, we identified 42 genes encoding flagellum‐associated proteins (Table S6).

Some Volvocine algae, including *Chlamydomonas*, have well‐documented heterothallism powered by sex‐determining regions located on UV sex chromosomes (Yamamoto *et al*., 2021). However, nothing is known about possible mating types in trebouxiophycean algae. We therefore searched the *T*. sp. ‘A48’ genome for mating‐type specific genes using sequences from *C. reinhardtii*, but failed to locate both MT+ specific *FUS1* and MT– specific *MTD1*. The genome encodes five RWP–RK transcription factor genes (Table S5), which are implicated in sex determination in diverse chlorophyte algae (Coelho *et al*., 2018), but their roles remain unclear.

### Genes involved in carbon‐concentrating mechanisms in *Trebouxia*

Carbon‐concentrating mechanisms (CCMs) provide a strategy for increasing photosynthesis efficiency. In land plants, CCM exists in the form of crassulacean acid metabolism (CAM) and C4 metabolism, while eukaryotic algae mostly possess CCMs based on active uptake of HCO_3_^−^ or CO_2_ (Maberly & Gontero, 2017). In chlorophyte algae, CCM relies on pyrenoids, which are organelles residing in chloroplasts. Not all lichen photobionts possess CCM, and their presence or absence can influence the geographic ranges of lichen species (Koch *et al*., 2023). Pyrenoids are present in *Trebouxia* algae, and their morphology has long been used as a diagnostic trait for distinguishing between different species (Bordenave *et al*., 2022). In the genome of *Trebouxia*, we found both key gene groups involved in pyrenoid‐based CCM (He *et al*., 2023): bestrophin‐like channels, which transport HCO_3_^−^ into the thylakoid lumen, and carbonic anhydrases, which catalyze conversion between CO_2_ and HCO_3_^−^ (Table S6). Three putative bestrophin‐like channel genes (GTX0536PRED_011525‐T1, GTX0536PRED_011526‐T1, and GTX0536PRED_011527‐T1) were located next to each other on contig GTX0536_17, separated by only 1534 and 2539 bp, respectively. While two modes of CCM are identified in *Chlamydomonas reinhardtii*, we were able to find genes associated with only one. Namely, active chloroplast HCO_3_^−^ uptake, which in *C. reinhardtii* only occurs in very low CO_2_ conditions (He *et al*., 2023). In the *Trebouxia* genome, we found genes similar to transporters *HLA3* and *LCIA*, which move HCO_3_^−^ across the plasma membrane and the chloroplast envelope, respectively (Table S6).

In addition, we found multiple genes involved in C4 metabolism, the CCM of land plants. These genes included phosphoenolpyruvate carboxylase (*PEPC*), phosphoenolpyruvate carboxykinase (*PCK*), and others (Fig. S2), consistent with a previous report on *Trebouxia* (Alberola, 2015). However, these genes are widespread across many eukaryotes, including nonphotosynthetic lineages, and their presence alone cannot indicate C4 metabolism (Chi *et al*., 2014).

### Horizontally transferred genes in *Trebouxia*

**Fig. 4:**
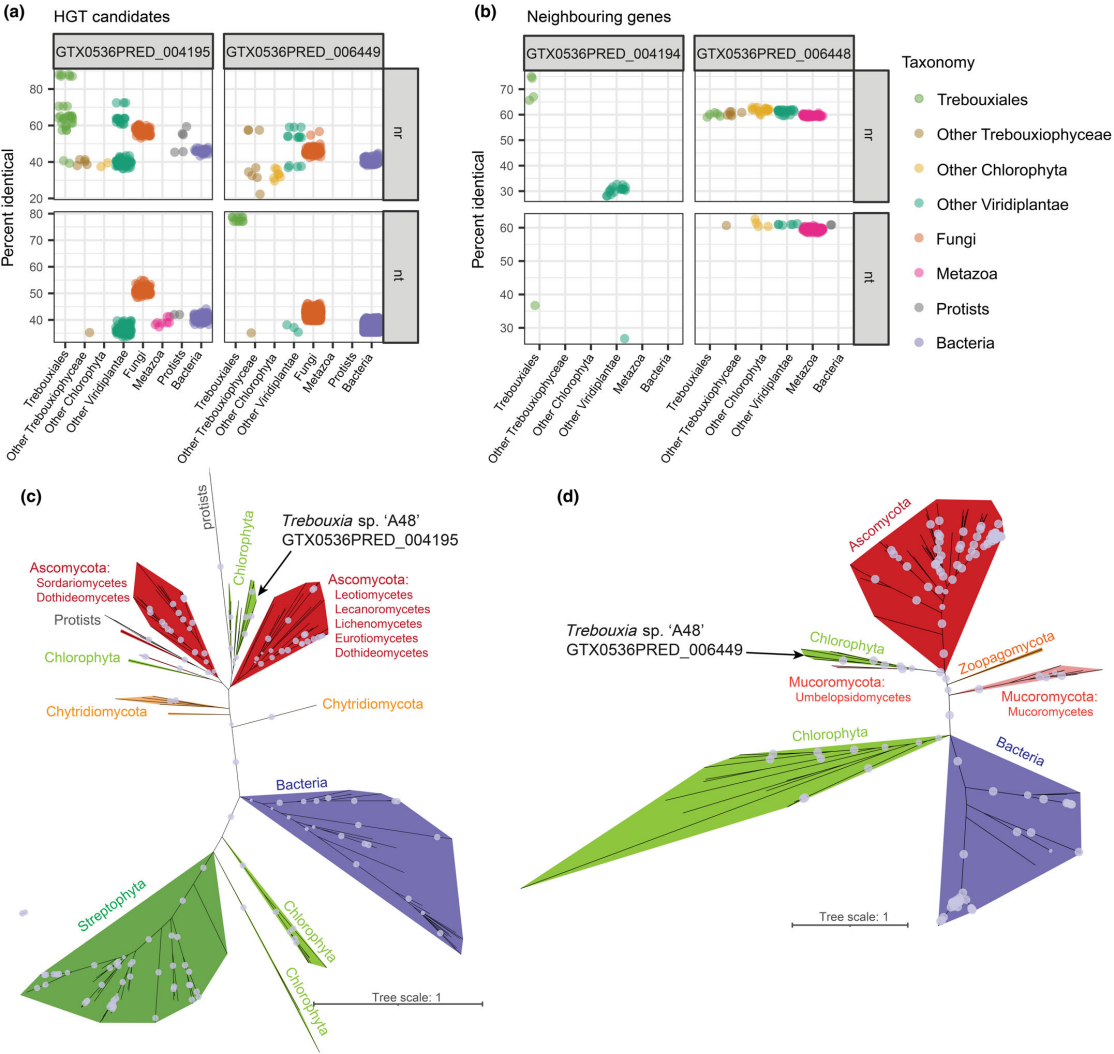
Predicted instances of horizontal gene transfer (HGT) in the *Trebouxia* sp. ‘A48’ genome. (a, b) Results of a BLAST search to the NCBI nt and nr databases for the HGT candidates (a) and their neighboring genes (b). Each dot represents a hit, positioned based on the taxonomy of the subject sequence and the percent identity of the hit. Only hits with the query coverage above 75% are shown. (c, d) Phylogenetic trees derived from a maximum‐likelihood analysis of homologues for the HGT candidates GTX0536PRED_004195 (c) and GTX0536PRED_006449 (d). Dots represent bootstrap support > 90%. The trees in Newick format can be found in GitHub (https://github.com/metalichen/2025‐Trebouxia‐genome). The sequences used in the analysis are listed in Supporting Information Table S7.

We confirmed two instances of ancient horizontal gene transfer (HGT) from fungi to the *T*. sp. ‘A48’ genome. Previously, Beck *et al*. (2015) reported three putative HGT candidates from a *T. decolorans* genome. We were able to find two of them in the *T*. sp. A48 genome sequence: an oxidoreductase‐like gene (GTX0536PRED_004195) and a SLAC anion channel/TDT transporter‐like gene (GTX0536PRED_006449). Since many more algal genomes are available now compared with the date of the original publication, we decided to proof‐test the HGT reports. This yielded results largely consistent with Beck *et al*. (2015). When searched against the NCBI nr and nt databases, the sequences returned mostly fungal hits (Fig. 4a, Table S7). For comparison, we analyzed two genes immediately upstream of the HGT candidates, which returned no fungal hits at all (Fig. 4b). In the phylogenies, both candidate HGT genes were recovered in chlorophyte clades nested within the larger fungal clade (Fig. 4c,d). In both cases, the sequences were recovered not in the lecanoromycete clade, as would be expected for a mycobiont‐to‐photobiont HGT, but in the basal part of the fungal clade, which indicates a more ancient transfer. The genome also encodes two enzymes from the CAZy family GH8 (Table S5), which were recently revealed as potentially horizontally transferred to Trebouxiophyceae from bacteria (Puginier *et al*., 2024).

### Secretome of *T.* sp. ‘A48’ is enriched in hydrolases and redox enzymes

**Fig. 5:**
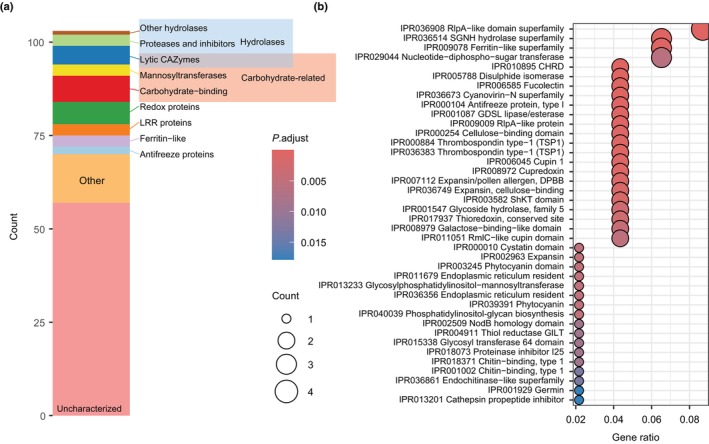
The predicted secretome of *Trebouxia* sp. ‘A48’. (a) Composition of the photobiont secretome. The 103 secreted genes were predicted *in silico* and grouped based on their functional annotation. LRR, leucine‐rich repeat; CAZyme, carbohydrate‐active enzyme. (b) Enrichment analysis showing InterProScan domains overrepresented in the secretome. The size of the dots represents the number of proteins; the color represents *P*_adj_‐value calculated using ClusterProfiler.

Algal secreted proteins have been implicated in both symbiosis and the desiccation response of Trebouxiophyceae algae (Armaleo *et al*., 2019; González‐Hourcade *et al*., 2021). Using three bioinformatics tools (SignalP, deep TMHMM, and WolfPSORT), we identified 103 proteins as being putatively secreted by *Trebouxia* (Fig. 5a). The secretome size is consistent with that reported from other Trebouxiophyceae (González‐Hourcade *et al*., 2021). Several functional groups were overrepresented in the secretome, including hydrolases and redox proteins (Fig. 5b).

Fungal lectins have long been hypothesized to play a role in lichen symbiont recognition and adhesion, but less is known regarding photobiont‐produced lectins (Singh & Walia, 2014). We found that carbohydrate‐related proteins, including enzymes, accounted for 17% of the predicted secretome. The secretome contained one endochitinase‐like glycoside hydrolase, for example, which might target fungal cell walls. Seven secreted proteins were also predicted to bind carbohydrates; those included three expansin‐like proteins, two of which also contained an RlpA‐like domain, a Ricin B lectin‐like and a fucolectin‐like protein. Three alpha‐mannosyltransferases from CAZy families GT32, GT64, and GT71 were also detected, which is notable given the abundance of alpha‐mannans in lichen thalli (Spribille *et al*., 2020).

As oxidative stress accompanies desiccation, lichen symbionts possess mechanisms for protection against reactive oxygen species (ROS) (Kranner *et al*., 2003). Among the redox proteins from lichen algae, one predicted secreted protein has previously been implicated in the desiccation response (González‐Hourcade *et al*., 2021). In the secretome, we identified several proteins similar to cupredoxin, thioredoxin, and manganese/iron superoxide dismutase (Table S8). The secretome also contained several germin‐like proteins, which might potentially be involved in ROS production. Whether these proteins are indeed involved in ROS production or response, or play another role, remains unclear. Notably, the secretome also contained two proteins annotated as antifreeze protein 1‐like (Fig. 5). This functional family was originally described from marine animals, and while similar proteins appear in chlorophyte algae (e.g. Cre09.g398150 in *C. reinhardtii*), their role remains unknown.

Among the functionally characterized secreted proteins, many shared annotations with gene families identified by Puginier *et al*. (2024) as associated with lichenization in chlorophyte algae. These included ferritin, fucolectin, and cupin‐like proteins. In general, the contents of the predicted secretome appeared rather conserved when compared with other *Trebouxia* species. Nearly half of the predicted secreted proteins (*n* = 46), for example, were encoded by genes from orthogroups shared between all eight surveyed genomes. The conserved portion of the secretome included 59% of all functionally characterized proteins and all but one predicted hydrolase. By contrast, nine proteins were encoded by genes from orthogroups unique to *T*. sp. ‘A48’, only one of them had a leucine‐rich repeat domain, and the rest lacked functional annotation. One additional secreted protein belonged to an orthogroup unique to *T. decolorans* species complex; it too had no functional annotation.

More than half of the predicted secreted proteins (*n* = 53) lacked any functional annotation that we could assign using standard databases, such as CAZy, MEROPS, UniProt, Pfam, or InterProScan (Table S8). This problem occurs in studies of secretomes of various organisms, presumably due to a rapid evolution of secreted proteins (Jackson *et al*., 2006; Brown *et al*., 2012; Nogueira *et al*., 2012). The uncharacterized secreted proteins were shorter than average (Fig. S3a). Over 75% (*n* = 44) belonged to the category of small secreted proteins (SSPs), as their sequences were shorter than 300 amino acids. By contrast, proteins of < 300 amino acids accounted for 52% (*n* = 26) of annotated secreted proteins and only 38% of proteins across the predicted proteome. Twelve secreted proteins were cysteine‐rich (defined as > 5% cysteine), which is commonly observed in secreted proteins with antimicrobial functions (Silverstein *et al*., 2007). Two of the secreted cysteine‐rich proteins contained cellulose‐binding domains (IPR000254); the rest were uncharacterized (Fig. S3b). By searching the uncharacterized secreted proteins against the Conserved Domain Database, we were able to add annotations to nine additional proteins, including hits to the Chi1 superfamily (chitinases) and Stig1 superfamily (a plant signaling protein, Table S8).

### Gene expression in lichen thalli and axenic culture

**Fig. 6:**
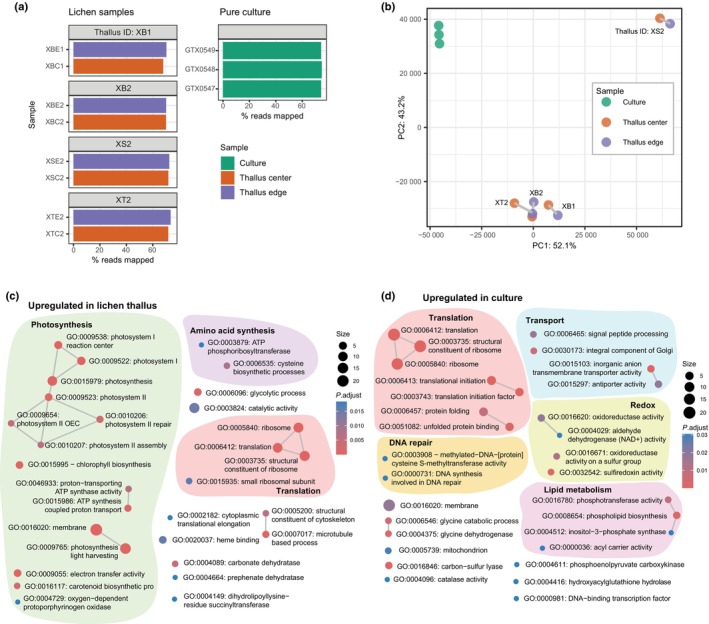
Transcriptional profile analysis of *Trebouxia* sp. ‘A48’ in lichen thalli and axenic culture. (a) The percentage of reads aligned to the reference in libraries from lichen thalli (produced by filtering metatranscriptomic libraries to include only algal reads) and from aposymbiotic pure culture. For the lichen thalli libraries, bars are grouped according to the thallus (individual) ID; the part of the thallus used for sequencing is shown in color. (b) Principal component analysis plot for RNA‐seq analysis based on transcript per million values. Samples are colored by sample type. For the lichen thalli libraries, samples originating from the thallus are connected with a line. (c, d) Enrichment plots showing Gene Ontology (GO) terms enriched in genes upregulated in: (c) lichen thalli; (d) pure aposymbiotic *T*. sp. ‘A48’ culture. The size of the node corresponds to the number of transcripts assigned to the GO term. The color shows the enrichment score (*P*_adj_‐value) calculated using ClusterProfiler. Two terms are linked when a single transcript is annotated with both terms.

To showcase the use of the genome of *T*. sp. ‘A48’ for lichen functional research, we used it as a reference in transcriptomic analysis. We compared algal gene expression in aposymbiotic cultures grown in nutrient‐rich medium and within symbiotic intact lichen thalli. We combined transcriptomic data produced *de novo* from cultures with the algal portion of metatranscriptomic libraries generated in our previous study (Tagirdzhanova *et al*., 2025). All libraries had a similar percentage of reads mapped to the reference (Fig. 6a). While the culture samples grouped together, samples produced from different lichen thalli differed substantially (Fig. 6b), mirroring previous observations of mycobiont gene expression (Tagirdzhanova *et al*., 2025).

We identified 755 DEGs, of which 570 were upregulated in culture and 185 in lichen thallus (Tables S9, S10). Transcripts assigned to Gene Ontology (GO) terms relating to translation were overrepresented among both culture and lichen thallus upregulated genes (Fig. 6c,d). Transcripts associated with photosynthesis were overrepresented in the lichen thallus‐upregulated list, consistent with our expectations: because the axenic culture was grown on nutrient‐rich medium, suppression of photosynthesis would be expected from a mixotrophic alga. Two genes encoding pyrenoid‐associated carbonic anhydrases were also upregulated in lichen thalli. At the same time, the culture exhibited signs of stress, as DNA repair GO terms were overrepresented. This observation was also confirmed by culture‐overrepresented InterProScan domains, which included multiple heat shock proteins (Fig. S4). Five transcripts encoding putatively secreted proteins were differentially expressed: three, including a cupin, were upregulated in culture, and two, including a ferritin, were upregulated in lichen thalli. Only one flagellum‐associated gene was differentially expressed: GTX0536PRED_006965 (Intraflagellar transport protein 20) was upregulated in culture. Contrary to our expectations, genes associated with reproduction and cell division were not identified as statistically significantly upregulated in culture. At the same time, numerous meiosis‐associated genes, including meiotic nuclear division protein 1 and meiosis‐specific cohesion protein *REC8*, showed higher levels of expression in culture across all samples (Table S6). The meiotic recombination protein gene *DMC1* was expressed only in culture. The majority of flagellum‐associated genes showed low levels of expression in at least one lichen sample, suggesting that these genes might be expressed in nonflagellated cells as well.

Short‐chain dehydrogenase/reductases (SDRs) were previously linked to the biosynthesis of ribitol, the compound channeled from *Trebouxia* to the mycobiont (Puginier *et al*., 2024). Four SDRs were upregulated in the lichen thallus. Among the genes upregulated in the lichen thallus, we also found a protein with a sugar phosphate transporter domain (IPR004853). By contrast, among the culture‐upregulated genes, we found two putative sugar/polyol transporters (IPR003663) as well as two amino acid transporters and one ammonium transporter (Table S9). These transporters could be involved in nutrient uptake from the culture medium.

The lichen samples included in this analysis represent four pairs, each produced from an individual thallus and each including a sample from the older central part and the younger edge part of the thallus (Table S3). This sampling allowed us to compare gene expression between the edge and center while controlling for thallus identity. Perhaps owing to the great degree of variation between individual thalli, this analysis did not yield many DEGs. In total, we found four transcripts that were upregulated in the edge, although all showed low |b‐value| (analogous to log fold change) (Table S11). Notably, three out of four edge‐upregulated genes shared the same InterProScan annotation and putatively encoded Chl*a–b* binding proteins, which are a component of the light‐harvesting complex. The transcripts showed low sequence similarity, making artifacts of cross‐mapping unlikely, and overall levels of expression within the normal range (Fig. S5). In each sample pair, these genes were more highly expressed in the edge compared with the center.

## Discussion

Lichen symbioses constitute a wide array of associations between vastly different fungi and photosynthetic organisms united by a similar outcome in the complex form of a multicellular lichen thallus (Spribille *et al*., 2022). The body plans of lichen thalli are anatomically complex, stable, and consistently reproduce over generations, implying the existence of some form of developmental program. Yet, remarkably, they result from the association of morphologically simple fungal, cyanobacterial, or algal partners. The complexity of partners in lichens is also increasingly apparent with the consistent presence of bacteria and nonmycobiont fungi reported from several lichens, including *X. parietina* (Hodkinson & Lutzoni, 2009; Grimm *et al*., 2021; Leiva *et al*., 2021; Tagirdzhanova *et al*., 2024, 2025). However, the majority of biomass in a lichen is typically represented by a major mycobiont fungus and a photosynthetic partner. In this regard, *Trebouxia* algae play a prominent role in lichen biology as a whole because they participate in the most species‐rich groups of lichens, formed by mycobionts from orders Lecanorales, including Parmeliaceae, Caliciales, and Teloschistes, and are also paired with a diversity of mycobionts from classes Lecanoromycetes, Lichinomycetes, Arthoniomycetes, and Eurotiomycetes (Sanders & Masumoto, 2021). Investigating the biology of *Trebouxia* is therefore pivotal to understanding lichen physiology and development.

In this report, we generated a chromosome‐level assembly of the genome of *T*. sp ‘A48’ from the *T. decolorans* species complex (soon to be described as *T. tabarcae*; P. Moya, I. Garrido‐Benavent, pers. comm.). This group of species is primarily associated with the mycobionts of the Teloschistales order, but is also reported from lichens formed by Lecanorales and Caliciales fungi (Dal Grande *et al*., 2014; Werth & Sork, 2014). We used the genome to investigate the evolutionary history of the alga and its relationship with its fungal partner. We have confirmed two instances of ancient horizontal gene transfer from a fungal origin and also profiled the predicted secretome of the alga. We identified genes likely to play a role in carbon concentration – an important physiological mechanism that allows algae to withstand low CO_2_ conditions. CCM appears especially important for lichens exposed to direct light and liquid water (Koch *et al*., 2022), which is often the case for *X. parietina*. Some of the CCM‐associated genes were upregulated in the lichen thalli, consistent with higher rates of photosynthesis compared with growth in axenic culture, yet many have consistently high levels of expression across all studied samples. The presence of CCM in *Trebouxia* might therefore be one of the reasons for its prevalence in lichen symbioses.

Fungal secreted proteins play a crucial role in both mutualistic and parasitic relationships (Lowe & Howlett, 2012), and lichen fungi are likely no exception (Snelders *et al*., 2022; Tagirdzhanova *et al*., 2025). Predicted effector proteins are, for example, found in *X. parietina* and potentially deployed by the mycobiont during lichen development, perhaps to manipulate its photobiont partner. Much less is known, however, about the secretomes of chlorophyte algae, but existing studies suggest that algae secrete proteins through both conventional and unconventional exocytotic pathways, and that their secretomes are dominated by extracellular enzymes (Xiao & Zheng, 2016; Choi *et al*., 2021). A recent study used proteomics and genomics to profile secretomes of two lichen algae, including *T. lynnae* (González‐Hourcade *et al*., 2021). *T*. sp ‘A48’ has a similar secretome profile to *T. lynnae*, and both secretomes contain lectins and CAZymes potentially involved in symbiotic interactions or cell adhesion.

Our results also provide evidence that *T*. sp ‘A48’ is haploid and capable of sexual reproduction, as its genome encodes all key genes required for meiosis and mating. These genes appear to be expressed, and while many have higher levels of expression in culture, the majority showed at least low levels of expression in lichen thalli as well. At the same time, it is unclear to what extent sexual reproduction occurs in nature. Marker‐gene‐based analysis of the genetic structure of *X. parietina* photobionts showed low admixture rates, consistent with *T. decolorans* existing primarily as a clonal organism (Wyczanska *et al*., 2023). Notably, while examining the *T*. sp. ‘A48’ culture, we detected flagellated cells highly similar to those identified as gametes in *T. lynnae* (Gazquez *et al*., 2024) (Fig. 1b), which is consistent with the presence of flagellum‐associated genes in the *T*. sp. ‘A48’ genome. However, these cells might represent not gametes but asexual zoospores, as both types of flagellated cells are reported from *Trebouxia* (Boccato *et al*., 2025). In the absence of flow cytometry data, our results should be treated as preliminary with regard to the ploidy of this strain.

Provided with the newly produced genome of the *X. parietina* photobiont, we were able to analyze the algal component of *X. parietina* metatranscriptomic data that we generated previously (Tagirdzhanova *et al*., 2025). We compared algal gene expression in intact lichen thalli to aposymbiotic axenic algal cultures growing on nutrient‐rich media typically used for culturing *Trebouxia*, as *Trebouxia* has long been known as a mixotroph (Fox, 1967). In our data, the switch to heterotrophic growth is reflected in the suppression of photosynthesis‐related genes that we observed in culture. Notably, some of the same photosynthesis‐related genes were also upregulated in *Trebouxia* in the younger, actively growing part of the lichen thallus compared with the older central part. The high degree of variation between individual thalli has complicated our analysis but did highlight the likely difference in photosynthetic activity. Several genes from the group of SDR enzymes previously linked to ribitol biosynthesis (Puginier *et al*., 2024) were upregulated in lichen thalli, which can be seen as indirect evidence confirming their role in the lichen symbiosis. Future work on more targeted profiling of gene expression in different parts of a lichen thallus might shed light on the photobiont contribution to symbiotic architectures and the mechanisms preventing division of photobiont cells in older, mature parts of a lichen body (Honegger, 1993).

HGT has been posited as one of the drivers of lichenization in green algae (Puginier *et al*., 2024; Mariault *et al*., 2025). Notably, one of the identified horizontally transferred genes, GTX0536PRED_004195, belongs to the same group of SDR enzymes linked to ribitol biosynthesis. This gene was expressed at variably high levels in samples from both culture and lichen thalli, yet we cannot rule out that it plays a role in the symbiotic interactions. The other gene, GTX0536PRED_006449, is an anion channel with unknown function. By contrast, it was highly expressed in culture and missing from lichen thalli. Similar anion channels are present in various groups within the green lineage, including chlorophyte algae, and are linked to abiotic stress response (Jiang *et al*., 2022). Given that the gene expression of the samples from culture showed other signs of stress, we can hypothesize that GTX0536PRED_006449 plays a role in allowing the alga to withstand exposure to harsh conditions.

Arguably, the main obstacle to understanding the lichen symbiosis is our inability to recreate the symbiosis from its constituent parts under controlled conditions. Lichen studies instead most often rely on natural lichen thalli collected from the field, which inevitably introduces a wide array of variables beyond researchers' control. One such factor is the identity of symbionts, which can vary between individual lichen thalli and often resembles a rotating cast of players rather than a straightforward one‐to‐one relationship (Spribille *et al*., 2022). Lichen mycobionts tend, for example, to associate with several closely related photobiont lineages, while one algal genus often associates with a wider variety of mycobionts (Sanders & Masumoto, 2021). The mechanisms for lichen symbiont recognition, however, remain largely unknown. In this study, we compared the genomes of two *Trebouxia* photobionts of *X. parietina* lichens. Despite belonging to different candidate species, the two genomes were similar in both genomic structure and gene content. We attempted to identify the key features of the two genomes that distinguish them from other *Trebouxia* species that are not known from *Xanthoria* lichens. Among the genes present in both genomes but not found in any other studied *Trebouxia*, two candidates encoded proteins that can selectively bind polysaccharides, hinting at a potential role in symbiont recognition. Neither of these gene products was, however, identified as secreted, although it is possible that they are secreted through unconventional routes. Given that the *X. parietina* symbiosis is established *de novo* every generation from its constituent fungal and algal partners, the photobiont composition is remarkably consistent across the wide geographic area inhabited by this lichen. The ITS sequence of our UK‐originated strain was, for instance, nearly identical to those of *X. parietina* photobionts from France and Australia, and highly similar to those from North America. Given this degree of conservation, the genome sequence generated here may provide a valuable reference for studies on *X. parietina* regardless of the geographic location of collected lichens.

In summary, we set out to produce a reference genome for the photobiont of *X. parietina*, which can be considered a model lichen symbiosis. With an N50 of 3.7 Mbp and 16 contigs out of 20 likely representing telomere‐to‐telomere chromosome assemblies, this genome is, to our knowledge, the most contiguous and complete genome of a lichen alga currently available. The corresponding algal strain is available to the community and can be obtained via the SAG algal culture collection as SAG 2671. We hope that together these resources will aid research into chlorophyte algae genomics and evolution, as well as experimental studies of the lichen symbiosis.

## Competing interests

None declared.

## Author contributions

GT and NJT planned and designed the study. JR and GT performed lab work. GT performed bioinformatic analysis and microscopy and drafted the manuscript. All authors contributed to editing.

## Disclaimer

The New Phytologist Foundation remains neutral with regard to jurisdictional claims in maps and in any institutional affiliations.

## Supporting information


**Fig. S1** Assembly of *Trebouxia* sp. ‘A48’ genome.
**Fig. S2** Metabolic map showing genes involved in carbon fixation and C4 metabolism.
**Fig. S3** Uncharacterized and characterized portions of the secretome in comparison to the proteome.
**Fig. S4** Enrichment plots showing InterProScan domains enriched in genes upregulated in: A. Lichen thalli. B. pure *Trebouxia* sp. ‘A48’ culture.
**Fig. S5** Differential gene expression between thallus parts.

**Table S1** ITS sequences used for phylogenetic tree.
**Table S2** Genomes used in the phylogenomic tree.
**Table S3** Transcriptomic samples included in the differential gene expression analysis.
**Table S4** Genes assigned to the orthogroups unique to the two genomes of *Xanthoria parietina* photobionts.
**Table S5** Genome annotation table produced by Funannotate.
**Table S6** Genes belonging to functional groups of interest: meiosis and sexual reproduction machinery, flagellum, and carbon‐concentrating associated genes.
**Table S7** Results of BLAST search against the NCBI nt and nr databases for the two candidate HGT genes.
**Table S8** Predicted secretome of *Trebouxia* sp. ‘A48’.
**Table S9** Differentially expressed genes in the comparison between the algal culture and intact lichen thallus.
**Table S10** Output of kallisto for *Trebouxia* sp. ‘A48’ gene expression in all samples.
**Table S11** Differentially expressed genes in the comparison between the thallus edge and center.
Please note: Wiley is not responsible for the content or functionality of any Supporting Information supplied by the authors. Any queries (other than missing material) should be directed to the *New Phytologist* Central Office.

## Data Availability

The genome assembly and annotation and the raw data are publicly available (ENA project: PRJEB95800, WGS Sequence Set: CBIHSB010000000.1). All scripts created for this project and detailed information on the usage of bioinformatics software are available at GitHub (https://github.com/metalichen/2025‐Trebouxia‐genome).

## References

[nph70728-bib-0001] Ahmadjian V . 2002. Trebouxia: reflections on a perplexing and controversial lichen photobiont. In: Seckbach J , ed. Symbiosis: mechanisms and model systems. Cellular origin and life in extreme habitats. Dordrecht, the Netherlands; Boston, MA, USA; London, UK: Kluwer Academic, 375–383.

[nph70728-bib-0002] Alberola FM . 2015. Caracterización genómica del microalga *Trebouxia* sp.TR9 aislada de liquen *Ramalina farinacea* (L.) Ach. mediante secuenciación masiva. Doctoral dissertation, Universitat de València.

[nph70728-bib-0003] Almagro Armenteros JJ , Tsirigos KD , Sønderby CK , Petersen TN , Winther O , Brunak S , von Heijne G , Nielsen H . 2019. SignalP 5.0 improves signal peptide predictions using deep neural networks. Nature Biotechnology 37: 420–423. 10.1038/s41587-019-0036-z 30778233

[nph70728-bib-0004] Almer J , Resl P , Gudmundsson H , Warshan D , Andrésson ÓS , Werth S . 2023. Symbiont‐specific responses to environmental cues in a threesome lichen symbiosis. Molecular Ecology 32: 1045–1061. 36478478 10.1111/mec.16814

[nph70728-bib-0005] Ament‐Velásquez SL , Tuovinen V , Bergström L , Spribille T , Vanderpool D , Nascimbene J , Yamamoto Y , Thor G , Johannesson H . 2021. The plot thickens: haploid and triploid‐like thalli, hybridization, and biased mating type ratios. Frontiers in Fungal Biology 2: 656386. 37744149 10.3389/ffunb.2021.656386 PMC10512270

[nph70728-bib-0006] Armaleo D , Müller O , Lutzoni F , Andrésson ÓS , Blanc G , Bode HB , Collart FR , Dal Grande F , Dietrich F , Grigoriev IV *et al*. 2019. The lichen symbiosis re‐viewed through the genomes of *Cladonia grayi* and its algal partner *Asterochloris glomerata* . BMC Genomics 20: 605. 31337355 10.1186/s12864-019-5629-x PMC6652019

[nph70728-bib-0007] Barreno E , Muggia L , Chiva S , Molins A , Bordenave C , García‐Breijo F , Moya P . 2022. *Trebouxia lynnae* sp. nov. (Former Trebouxia sp. TR9): biology and biogeography of an epitome lichen symbiotic microalga. Biology 11: 1196. 36009823 10.3390/biology11081196 PMC9405249

[nph70728-bib-0008] Beck A , Divakar PK , Zhang N , Molina MC , Struwe L . 2015. Evidence of ancient horizontal gene transfer between fungi and the terrestrial alga Trebouxia. Organisms, Diversity and Evolution 15: 235–248.

[nph70728-bib-0009] Boccato E , Porrelli D , Ametrano CG , Carniel FC , Tretiach M . 2025. Zoospore diversity and sexual reproduction in the lichen‐forming genus Trebouxia: from neglected evidence to new facts. Plant Biology 27: 1137–1149. 40462319 10.1111/plb.70042 PMC12477308

[nph70728-bib-0010] Bordenave CD , Muggia L , Chiva S , Leavitt SD , Carrasco P , Barreno E . 2022. Chloroplast morphology and pyrenoid ultrastructural analyses reappraise the diversity of the lichen phycobiont genus Trebouxia (Chlorophyta). Algal Research 61: 102561.

[nph70728-bib-0011] Bray NL , Pimentel H , Melsted P , Pachter L . 2016. Near‐optimal probabilistic RNA‐seq quantification. Nature Biotechnology 34: 525–527. 10.1038/nbt.3519 27043002

[nph70728-bib-0012] Brown NA , Antoniw J , Hammond‐Kosack KE . 2012. The predicted secretome of the plant pathogenic fungus *Fusarium graminearum*: a refined comparative analysis. PLoS ONE 7: e33731. 22493673 10.1371/journal.pone.0033731 PMC3320895

[nph70728-bib-0013] Camacho C , Coulouris G , Avagyan V , Ma N , Papadopoulos J , Bealer K , Madden TL . 2009. BLAST+: architecture and applications. BMC Bioinformatics 10: 421. 20003500 10.1186/1471-2105-10-421 PMC2803857

[nph70728-bib-0014] Capella‐Gutiérrez S , Silla‐Martínez JM , Gabaldón T . 2009. trimAl: a tool for automated alignment trimming in large‐scale phylogenetic analyses. Bioinformatics 25: 1972–1973. 19505945 10.1093/bioinformatics/btp348 PMC2712344

[nph70728-bib-0015] Carniel FC , Gerdol M , Montagner A , Banchi E , De Moro G , Manfrin C , Muggia L , Pallavicini A , Tretiach M . 2016. New features of desiccation tolerance in the lichen photobiont *Trebouxia gelatinosa* are revealed by a transcriptomic approach. Plant Molecular Biology 91: 319–339. 26992400 10.1007/s11103-016-0468-5

[nph70728-bib-0016] Chan PP , Lowe TM . 2019. tRNAscan‐SE: searching for tRNA genes in genomic sequences. Methods in Molecular Biology 1962: 1–14. 31020551 10.1007/978-1-4939-9173-0_1 PMC6768409

[nph70728-bib-0017] Chavarria‐Pizarro T , Resl P , Janjic A , Werth S . 2022. Gene expression responses to thermal shifts in the endangered lichen *Lobaria pulmonaria* . Molecular Ecology 31: 839–858. 34784096 10.1111/mec.16281

[nph70728-bib-0018] Cheng H , Concepcion GT , Feng X , Zhang H , Li H . 2021. Haplotype‐resolved *de novo* assembly using phased assembly graphs with hifiasm. Nature Methods 18: 170–175. 33526886 10.1038/s41592-020-01056-5 PMC7961889

[nph70728-bib-0019] Chi S , Wu S , Yu J , Wang X , Tang X , Liu T . 2014. Phylogeny of C_4_‐photosynthesis enzymes based on algal transcriptomic and genomic data supports an archaeal/proteobacterial origin and multiple duplication for most C_4_‐related genes. PLoS ONE 9: e110154. 25313828 10.1371/journal.pone.0110154 PMC4196954

[nph70728-bib-0020] Chiva S , Bordenave CD , Assunção MFG , Craveiro SC , Calado AJ , Barreno E , Santos LMA . 2025. Revisiting the free‐living *Trebouxia* strains of the Coimbra Collection of Algae (ACOI): *Trebouxia valentina* sp. nov. (Trebouxiophyceae, Chlorophyta). European Journal of Phycology 60: 53–66.

[nph70728-bib-0021] Choi J , Shin J‐H , An HJ , Oh MJ , Kim S‐R . 2021. Analysis of secretome and N‐glycosylation of Chlorella species. Algal Research 59: 102466.

[nph70728-bib-0022] Coelho SM , Gueno J , Lipinska AP , Cock JM , Umen JG . 2018. UV chromosomes and haploid sexual systems. Trends in Plant Science 23: 794–807. 30007571 10.1016/j.tplants.2018.06.005 PMC6128410

[nph70728-bib-0023] Dal Grande F , Alors D , Divakar PK , Bálint M , Crespo A , Schmitt I . 2014. Insights into intrathalline genetic diversity of the cosmopolitan lichen symbiotic green alga *Trebouxia decolorans* Ahmadjian using microsatellite markers. Molecular Phylogenetics and Evolution 72: 54–60. 24412431 10.1016/j.ympev.2013.12.010

[nph70728-bib-0024] Dal Grande F , Beck A , Singh G , Schmitt I . 2013. Microsatellite primers in the lichen symbiotic alga *Trebouxia decolorans* (Trebouxiophyceae). Applications in Plant Sciences 1: 1200400. 10.3732/apps.1200400 PMC4105286 25202529

[nph70728-bib-0025] Del Campo EM , Gasulla F , Hell AF , González‐Hourcade M , Casano LM . 2023. Comparative transcriptomic and proteomic analyses provide new insights into the tolerance to cyclic dehydration in a lichen phycobiont. Microbial Ecology 86: 1725–1739. 37039841 10.1007/s00248-023-02213-x PMC10497648

[nph70728-bib-0026] Dobin A , Davis CA , Schlesinger F , Drenkow J , Zaleski C , Jha S , Batut P , Chaisson M , Gingeras TR . 2013. STAR: ultrafast universal RNA‐seq aligner. Bioinformatics 29: 15–21. 23104886 10.1093/bioinformatics/bts635 PMC3530905

[nph70728-bib-0027] Emms DM , Kelly S . 2019. OrthoFinder: phylogenetic orthology inference for comparative genomics. Genome Biology 20: 238. 31727128 10.1186/s13059-019-1832-y PMC6857279

[nph70728-bib-0028] Fernández‐Mendoza F , Domaschke S , García MA , Jordan P , Martín MP , Printzen C . 2011. Population structure of mycobionts and photobionts of the widespread lichen *Cetraria aculeata* . Molecular Ecology 20: 1208–1232. 21324011 10.1111/j.1365-294X.2010.04993.x

[nph70728-bib-0111] Flynn JM , Hubley R , Goubert C , Rosen J , Clark AG , Feschotte C , Smit AF . 2020. repeatmodeler2 for automated genomic discovery of transposable element families. Proceedings of the National Academy of Sciences, USA 117: 9451–9457. 10.1073/pnas.1921046117 PMC7196820 32300014

[nph70728-bib-0029] Fox CH . 1967. Studies of the cultural physiology of the lichen alga *Trebouxia* . Physiologia Plantarum 20: 251–262.

[nph70728-bib-0030] Fučíková K , Pažoutová M , Rindi F . 2015. Meiotic genes and sexual reproduction in the green algal class Trebouxiophyceae (Chlorophyta). Journal of Phycology 51: 419–430. 26986659 10.1111/jpy.12293

[nph70728-bib-0031] Garrido‐Benavent I , Chiva S , Bordenave CD , Molins A , Barreno E . 2022. *Trebouxia maresiae* sp. nov. (Trebouxiophyceae, Chlorophyta), a new lichenized species of microalga found in coastal environments. Cryptogamie Algologie 43: 135–145.

[nph70728-bib-0032] Gazquez A , Bordenave CD , Montero‐Pau J , Pérez‐Rodrigo M , Marco F , Martínez‐Alberola F , Muggia L , Barreno E , Carrasco P . 2024. From spores to gametes: a sexual life cycle in a symbiotic *Trebouxia microalga* . Algal Research 84: 103744.

[nph70728-bib-0033] González‐Hourcade M , Del Campo EM , Casano LM . 2021. The under‐explored extracellular proteome of aero‐terrestrial microalgae provides clues on different mechanisms of desiccation tolerance in non‐model organisms. Microbial Ecology 81: 437–453. 32989484 10.1007/s00248-020-01604-8

[nph70728-bib-0034] Greiner S , Lehwark P , Bock R . 2019. OrganellarGenomeDRAW (OGDRAW) version 1.3.1: expanded toolkit for the graphical visualization of organellar genomes. Nucleic Acids Research 47: W59–W64. 30949694 10.1093/nar/gkz238 PMC6602502

[nph70728-bib-0035] Greshake Tzovaras B , Segers FHID , Bicker A , Dal Grande F , Otte J , Anvar SY , Hankeln T , Schmitt I , Ebersberger I . 2020. What is in *Umbilicaria pustulata*? A metagenomic approach to reconstruct the holo‐genome of a lichen. Genome Biology and Evolution 12: 309–324. 32163141 10.1093/gbe/evaa049 PMC7186782

[nph70728-bib-0036] Grimm M , Grube M , Schiefelbein U , Zühlke D , Bernhardt J , Riedel K . 2021. The lichens' microbiota, still a mystery? Frontiers in Microbiology 12: 623839. 33859626 10.3389/fmicb.2021.623839 PMC8042158

[nph70728-bib-0037] Haas BJ , Salzberg SL , Zhu W , Pertea M , Allen JE , Orvis J , White O , Buell CR , Wortman JR . 2008. Automated eukaryotic gene structure annotation using EVidenceModeler and the Program to Assemble Spliced Alignments. Genome Biology 9: R7. 18190707 10.1186/gb-2008-9-1-r7 PMC2395244

[nph70728-bib-0038] Hallgren J , Tsirigos KD , Pedersen MD , Almagro Armenteros JJ , Marcatili P , Nielsen H , Krogh A , Winther O . 2022. DeepTMHMM predicts alpha and beta transmembrane proteins using deep neural networks. *bioRxiv* doi: 10.1101/2022.04.08.487609.

[nph70728-bib-0039] He S , Crans VL , Jonikas MC . 2023. The pyrenoid: the eukaryotic CO_2_‐concentrating organelle. Plant Cell 35: 3236–3259. 37279536 10.1093/plcell/koad157 PMC10473226

[nph70728-bib-0040] Hiltunen M , Ament‐Velásquez SL , Johannesson H . 2021. The assembled and annotated genome of the fairy‐ring fungus *Marasmius oreades* . Genome Biology and Evolution 13: evab126. 34051082 10.1093/gbe/evab126 PMC8290104

[nph70728-bib-0041] Hodkinson BP , Lutzoni F . 2009. A microbiotic survey of lichen‐associated bacteria reveals a new lineage from the Rhizobiales. Symbiosis 49: 163–180.

[nph70728-bib-0042] Honegger R . 1993. Developmental biology of lichens. New Phytologist 125: 659–677. 33874446 10.1111/j.1469-8137.1993.tb03916.x

[nph70728-bib-0043] Honegger R . 1996. Experimental studies of growth and regenerative capacity in the foliose lichen *Xanthoria parietina* . New Phytologist 133: 573–581.

[nph70728-bib-0044] Honegger R , Zippler U , Scherrer S , Dyer PS . 2004. Genetic diversity in *Xanthoria parietina* (L.) Th. Fr. (lichen‐forming ascomycete) from worldwide locations. Lichenologist 36: 381–390.

[nph70728-bib-0045] Horton P , Park K‐J , Obayashi T , Fujita N , Harada H , Adams‐Collier CJ , Nakai K . 2007. WoLF PSORT: protein localization predictor. Nucleic Acids Research 35: W585–W587. 17517783 10.1093/nar/gkm259 PMC1933216

[nph70728-bib-0047] Jackson DJ , McDougall C , Green K , Simpson F , Wörheide G , Degnan BM . 2006. A rapidly evolving secretome builds and patterns a sea shell. BMC Biology 4: 40. 17121673 10.1186/1741-7007-4-40 PMC1676022

[nph70728-bib-0048] Jiang W , Tong T , Chen X , Deng F , Zeng F , Pan R , Zhang W , Chen G , Chen ZH . 2022. Molecular response and evolution of plant anion transport systems to abiotic stress. Plant Molecular Biology 110: 397–412. 34846607 10.1007/s11103-021-01216-x

[nph70728-bib-0049] Kang DD , Li F , Kirton E , Thomas A , Egan R , An H , Wang Z . 2019. MetaBAT 2: an adaptive binning algorithm for robust and efficient genome reconstruction from metagenome assemblies. PeerJ 7: e7359. 31388474 10.7717/peerj.7359 PMC6662567

[nph70728-bib-0050] Katoh K , Standley DM . 2013. MAFFT multiple sequence alignment software version 7: improvements in performance and usability. Molecular Biology and Evolution 30: 772–780. 23329690 10.1093/molbev/mst010 PMC3603318

[nph70728-bib-0051] Knaus BJ , Grünwald NJ . 2017. vcfr: a package to manipulate and visualize variant call format data in R. Molecular Ecology Resources 17: 44–53. 27401132 10.1111/1755-0998.12549

[nph70728-bib-0052] Koboldt DC , Zhang Q , Larson DE , Shen D , McLellan MD , Lin L , Miller CA , Mardis ER , Ding L , Wilson RK . 2012. VarScan 2: somatic mutation and copy number alteration discovery in cancer by exome sequencing. Genome Research 22: 568–576. 22300766 10.1101/gr.129684.111 PMC3290792

[nph70728-bib-0053] Koch NM , Lendemer JC , Manzitto‐Tripp EA , McCain C , Stanton DE . 2023. Carbon‐concentrating mechanisms are a key trait in lichen ecology and distribution. Ecology 104: e4011. 36814365 10.1002/ecy.4011

[nph70728-bib-0054] Koch NM , Stanton D , Müller SC , Duarte L , Spielmann AA , Lücking R . 2022. Nuanced qualitative trait approaches reveal environmental filtering and phylogenetic constraints on lichen communities. Ecosphere 13: e4042.

[nph70728-bib-0055] Kono M , Kon Y , Ohmura Y , Satta Y , Terai Y . 2020. In vitro resynthesis of lichenization reveals the genetic background of symbiosis‐specific fungal‐algal interaction in *Usnea hakonensis* . BMC Genomics 21: 671. 32993496 10.1186/s12864-020-07086-9 PMC7526373

[nph70728-bib-0112] Kono M , Tanabe H , Ohmura Y , Satta Y , Terai Y . 2017. Physical contact and carbon transfer between a lichen‐forming *Trebouxia* alga and a novel *Alphaproteobacterium* . Microbiology 163: 678–691. 28535846 10.1099/mic.0.000461

[nph70728-bib-0056] Kopylova E , Noé L , Touzet H . 2012. SortMeRNA: fast and accurate filtering of ribosomal RNAs in metatranscriptomic data. Bioinformatics 28: 3211–3217. 23071270 10.1093/bioinformatics/bts611

[nph70728-bib-0057] Korf I . 2004. Gene finding in novel genomes. BMC Bioinformatics 5: 59. 15144565 10.1186/1471-2105-5-59 PMC421630

[nph70728-bib-0058] Korhonen P , Kallio P . 1987. The effect of different night conditions on the CO_2_ fixation in a lichen *Xanthoria parietina* . Photosynthesis Research 12: 3–11. 24435576 10.1007/BF00019146

[nph70728-bib-0059] Kranner I , Zorn M , Turk B , Wornik S , Beckett RP , Batič F . 2003. Biochemical traits of lichens differing in relative desiccation tolerance. New Phytologist 160: 167–176. 33873534 10.1046/j.1469-8137.2003.00852.x

[nph70728-bib-0060] Lang BF , Beck N , Prince S , Sarrasin M , Rioux P , Burger G . 2023. Mitochondrial genome annotation with MFannot: a critical analysis of gene identification and gene model prediction. Frontiers in Plant Science 14: 1222186. 37469769 10.3389/fpls.2023.1222186 PMC10352661

[nph70728-bib-0061] Leiva D , Fernández‐Mendoza F , Acevedo J , Carú M , Grube M , Orlando J . 2021. The bacterial community of the foliose macro‐lichen *Peltigera frigida* is more than a mere extension of the microbiota of the subjacent substrate. Microbial Ecology 81: 965–976. 33404820 10.1007/s00248-020-01662-y

[nph70728-bib-0062] Leksin I , Shelyakin M , Zakhozhiy I , Kozlova O , Beckett R , Minibayeva F . 2024. Ultraviolet‐induced melanisation in lichens: physiological traits and transcriptome profile. Physiologia Plantarum 176: e14512. 39221518 10.1111/ppl.14512

[nph70728-bib-0063] Letunic I , Bork P . 2024. Interactive Tree of Life (iTOL) v6: recent updates to the phylogenetic tree display and annotation tool. Nucleic Acids Research 52: W78–W82. 38613393 10.1093/nar/gkae268 PMC11223838

[nph70728-bib-0064] Li H . 2018. Minimap2: pairwise alignment for nucleotide sequences. Bioinformatics 34: 3094–3100. 29750242 10.1093/bioinformatics/bty191 PMC6137996

[nph70728-bib-0065] Li H , Handsaker B , Wysoker A , Fennell T , Ruan J , Homer N , Marth G , Abecasis G , Durbin R , 1000 Genome Project Data Processing Subgroup . 2009. The sequence Alignment/Map format and SAMtools. Bioinformatics 25: 2078–2079. 19505943 10.1093/bioinformatics/btp352 PMC2723002

[nph70728-bib-0066] Lomsadze A , Ter‐Hovhannisyan V , Chernoff YO , Borodovsky M . 2005. Gene identification in novel eukaryotic genomes by self‐training algorithm. Nucleic Acids Research 33: 6494–6506. 16314312 10.1093/nar/gki937 PMC1298918

[nph70728-bib-0067] Lowe RGT , Howlett BJ . 2012. Indifferent, affectionate, or deceitful: lifestyles and secretomes of fungi. PLoS Pathogens 8: e1002515. 22396640 10.1371/journal.ppat.1002515 PMC3291654

[nph70728-bib-0068] Maberly SC , Gontero B . 2017. Ecological imperatives for aquatic CO_2_‐concentrating mechanisms. Journal of Experimental Botany 68: 3797–3814. 28645178 10.1093/jxb/erx201

[nph70728-bib-0069] Majoros WH , Pertea M , Salzberg SL . 2004. TigrScan and GlimmerHMM: two open source ab initio eukaryotic gene‐finders. Bioinformatics 20: 2878–2879. 15145805 10.1093/bioinformatics/bth315

[nph70728-bib-0070] Mariault L , Puginier C , Keller J , El Baidouri M , Delaux PM . 2025. Mechanisms, detection, and impact of horizontal gene transfer in plant functional evolution. Plant Cell 37: koaf195. 40834228 10.1093/plcell/koaf195 PMC12448838

[nph70728-bib-0071] Martin M . 2011. Cutadapt removes adapter sequences from high‐throughput sequencing reads. EMBnet.Journal 17: 10.

[nph70728-bib-0072] McKenna V , Archibald JM , Beinart R , Dawson MN , Hentschel U , Keeling PJ , Lopez JV , Martín‐Durán JM , Petersen JM , Sigwart JD *et al*. 2024. The Aquatic Symbiosis Genomics Project: probing the evolution of symbiosis across the Tree of Life. Wellcome Open Research 6: 254. 40438199 10.12688/wellcomeopenres.17222.2 PMC12117321

[nph70728-bib-0073] Minh BQ , Schmidt HA , Chernomor O , Schrempf D , Woodhams MD , von Haeseler A , Lanfear R . 2020. IQ‐TREE 2: new models and efficient methods for phylogenetic inference in the genomic era. Molecular Biology and Evolution 37: 1530–1534. 32011700 10.1093/molbev/msaa015 PMC7182206

[nph70728-bib-0074] Mistry J , Chuguransky S , Williams L , Qureshi M , Salazar GA , Ell S , Paladin L , Raj S , Richardson LJ *et al*. 2021. Pfam: the protein families database in 2021. Nucleic Acids Research 49: D412–D419. 33125078 10.1093/nar/gkaa913 PMC7779014

[nph70728-bib-0075] Moriya Y , Itoh M , Okuda S , Yoshizawa AC , Kanehisa M . 2007. KAAS: an automatic genome annotation and pathway reconstruction server. Nucleic Acids Research 35: W182–W185. 17526522 10.1093/nar/gkm321 PMC1933193

[nph70728-bib-0076] Muggia L , Nelsen MP , Kirika PM , Barreno E , Beck A , Lindgren H , Lumbsch HT , Leavitt SD , Trebouxia Working Group . 2020. Formally described species woefully underrepresent phylogenetic diversity in the common lichen photobiont genus Trebouxia (Trebouxiophyceae, Chlorophyta): an impetus for developing an integrated taxonomy. Molecular Phylogenetics and Evolution 149: 106821. 32294545 10.1016/j.ympev.2020.106821

[nph70728-bib-0077] Nogueira T , Touchon M , Rocha EPC . 2012. Rapid evolution of the sequences and gene repertoires of secreted proteins in bacteria. PLoS ONE 7: e49403. 23189144 10.1371/journal.pone.0049403 PMC3506625

[nph70728-bib-0078] Nyati S , Scherrer S , Werth S , Honegger R . 2014. Green‐algal photobiont diversity (Trebouxia spp.) in representatives of Teloschistaceae (Lecanoromycetes, lichen‐forming ascomycetes). Lichenologist 46: 189–212.

[nph70728-bib-0079] Palmer JM , Stajich J . 2020. Funannotate v1.8.1: Eukaryotic genome annotation. *Zenodo* .

[nph70728-bib-0080] Paysan‐Lafosse T , Blum M , Chuguransky S , Grego T , Pinto BL , Salazar GA , Bileschi ML , Bork P , Bridge A , Colwell L *et al*. 2023. InterPro in 2022. Nucleic Acids Research 51: D418–D427. 36350672 10.1093/nar/gkac993 PMC9825450

[nph70728-bib-0081] Pazos T , Moya P , Garrido‐Benavent I , Bordenave CD , Gazquez A , Chiva S . 2025. *Trebouxia barrenoae* sp. nov. (Trebouxiophyceae, Chlorophyta), a new lichenized microalga widespread in temperate ecosystems. Fottea 25: 68–80.

[nph70728-bib-0082] Pimentel H , Bray NL , Puente S , Melsted P , Pachter L . 2017. Differential analysis of RNA‐seq incorporating quantification uncertainty. Nature Methods 14: 687–690. 28581496 10.1038/nmeth.4324

[nph70728-bib-0083] Poquita‐Du RC , Otte J , Calchera A , Schmitt I . 2024. Genome‐wide comparisons reveal extensive divergence within the lichen photobiont genus, Trebouxia. Genome Biology and Evolution 16: evae219. 39475309 10.1093/gbe/evae219 PMC11523091

[nph70728-bib-0084] Puginier C , Libourel C , Otte J , Skaloud P , Haon M , Grisel S , Petersen M , Berrin J‐G , Delaux P‐M , Dal Grande F *et al*. 2024. Phylogenomics reveals the evolutionary origins of lichenization in chlorophyte algae. Nature Communications 15: 4452. 10.1038/s41467-024-48787-z PMC11126685 38789482

[nph70728-bib-0085] Quigley S , Damas J , Larkin DM , Farré M . 2023. syntenyPlotteR: a user‐friendly R package to visualize genome synteny, ideal for both experienced and novice bioinformaticians. Bioinformatics Advances 3: vbad161. 38023328 10.1093/bioadv/vbad161 PMC10660287

[nph70728-bib-0086] Rawlings ND , Waller M , Barrett AJ , Bateman A . 2014. MEROPS: the database of proteolytic enzymes, their substrates and inhibitors. Nucleic Acids Research 42: D503–D509. 24157837 10.1093/nar/gkt953 PMC3964991

[nph70728-bib-0087] Richardson DH , Smith DC . 1968. Lichen physiology: IX. Carbohydrate movement from the Trebouxia symbiont of *Xanthoria aureola* to the fungus. New Phytologist 67: 61–68.

[nph70728-bib-0088] Sanders WB , Masumoto H . 2021. Lichen algae: the photosynthetic partners in lichen symbioses. Lichenologist 53: 347–393.

[nph70728-bib-0089] Scharnagl K , Tagirdzhanova G , Talbot NJ . 2023. The coming golden age for lichen biology. Current Biology: CB 33: R512–R518. 37279685 10.1016/j.cub.2023.03.054

[nph70728-bib-0090] Schneider CA , Rasband WS , Eliceiri KW . 2012. NIH Image to ImageJ: 25 years of image analysis. Nature Methods 9: 671–675. 22930834 10.1038/nmeth.2089 PMC5554542

[nph70728-bib-0091] Seppey M , Manni M , Zdobnov EM . 2019. BUSCO: assessing genome assembly and annotation completeness. Methods in Molecular Biology 1962: 227–245. 31020564 10.1007/978-1-4939-9173-0_14

[nph70728-bib-0092] Silverstein KAT , Moskal WA Jr , Wu HC , Underwood BA , Graham MA , Town CD , VandenBosch KA . 2007. Small cysteine‐rich peptides resembling antimicrobial peptides have been under‐predicted in plants. The Plant Journal 51: 262–280. 17565583 10.1111/j.1365-313X.2007.03136.x

[nph70728-bib-0093] Singh RS , Walia AK . 2014. Characteristics of lichen lectins and their role in symbiosis. Symbiosis 62: 123–134.

[nph70728-bib-0094] Snelders NC , Rovenich H , Thomma BPHJ . 2022. Microbiota manipulation through the secretion of effector proteins is fundamental to the wealth of lifestyles in the fungal kingdom. FEMS Microbiology Reviews 46: fuac022. 35604874 10.1093/femsre/fuac022 PMC9438471

[nph70728-bib-0095] Spribille T , Resl P , Stanton DE , Tagirdzhanova G . 2022. Evolutionary biology of lichen symbioses. New Phytologist 234: 1566–1582. 35302240 10.1111/nph.18048

[nph70728-bib-0096] Spribille T , Tagirdzhanova G , Goyette S , Tuovinen V , Case R , Zandberg WF . 2020. 3D biofilms: in search of the polysaccharides holding together lichen symbioses. FEMS Microbiology Letters 367: fnaa023. 32037451 10.1093/femsle/fnaa023 PMC7164778

[nph70728-bib-0097] Stanke M , Waack S . 2003. Gene prediction with a hidden Markov model and a new intron submodel. Bioinformatics 19(Suppl 2): ii215–ii225. 14534192 10.1093/bioinformatics/btg1080

[nph70728-bib-0098] Tagirdzhanova G , Saary P , Cameron ES , Allen CC , Garber AI , Escandón DD , Cook AT , Goyette S , Nogerius VT , Passo A *et al*. 2024. Microbial occurrence and symbiont detection in a global sample of lichen metagenomes. PLoS Biology 22: e3002862. 39509454 10.1371/journal.pbio.3002862 PMC11542873

[nph70728-bib-0099] Tagirdzhanova G , Scharnagl K , Sahu N , Yan X , Bucknell A , Bentham AR , Jégousse C , Ament‐Velásquez SL , Onuț‐Brännström I , Johannesson H *et al*. 2025. Complexity of the lichen symbiosis revealed by metagenome and transcriptome analysis of *Xanthoria parietina* . Current Biology: CB 35: 799–817. 39889699 10.1016/j.cub.2024.12.041

[nph70728-bib-0100] Tillich M , Lehwark P , Pellizzer T , Ulbricht‐Jones ES , Fischer A , Bock R , Greiner S . 2017. GeSeq – versatile and accurate annotation of organelle genomes. Nucleic Acids Research 45: W6–W11. 28486635 10.1093/nar/gkx391 PMC5570176

[nph70728-bib-0101] UniProt Consortium . 2023. UniProt: the Universal Protein Knowledgebase in 2023. Nucleic Acids Research 51: D523–D531. 36408920 10.1093/nar/gkac1052 PMC9825514

[nph70728-bib-0102] Wang J , Chitsaz F , Derbyshire MK , Gonzales NR , Gwadz M , Lu S , Marchler GH , Song JS , Thanki N , Yamashita RA *et al*. 2023. The conserved domain database in 2023. Nucleic Acids Research 51: D384–D388. 36477806 10.1093/nar/gkac1096 PMC9825596

[nph70728-bib-0103] Werth S , Sork VL . 2014. Ecological specialization in Trebouxia (Trebouxiophyceae) photobionts of *Ramalina menziesii* (Ramalinaceae) across six range‐covering ecoregions of western North America. American Journal of Botany 101: 1127–1140. 25016008 10.3732/ajb.1400025

[nph70728-bib-0104] Wyczanska M , Wacker K , Dyer PS , Werth S . 2023. Local‐scale panmixia in the lichenized fungus *Xanthoria parietina* contrasts with substantial genetic structure in its *Trebouxia* photobionts. Lichenologist 55: 69–79.

[nph70728-bib-0105] Xiao R , Zheng Y . 2016. Overview of microalgal extracellular polymeric substances (EPS) and their applications. Biotechnology Advances 34: 1225–1244. 27576096 10.1016/j.biotechadv.2016.08.004

[nph70728-bib-0106] Xu M , De Boer H , Olafsdottir ES , Omarsdottir S , Heidmarsson S . 2020. Phylogenetic diversity of the lichenized algal genus *Trebouxia* (Trebouxiophyceae, Chlorophyta): a new lineage and novel insights from fungal‐algal association patterns of Icelandic cetrarioid lichens (Parmeliaceae, Ascomycota). Botanical Journal of the Linnean Society. Linnean Society of London 194: 460–468.

[nph70728-bib-0107] Yamamoto K , Hamaji T , Kawai‐Toyooka H , Matsuzaki R , Takahashi F , Nishimura Y , Kawachi M , Noguchi H , Minakuchi Y , Umen JG *et al*. 2021. Three genomes in the algal genus reveal the fate of a haploid sex‐determining region after a transition to homothallism. Proceedings of the National Academy of Sciences, USA 118: e2100712118. 10.1073/pnas.2100712118 PMC8166075 34011609

[nph70728-bib-0108] Yin Y , Mao X , Yang J , Chen X , Mao F , Xu Y . 2012. dbCAN: a web resource for automated carbohydrate‐active enzyme annotation. Nucleic Acids Research 40: W445–W451. 22645317 10.1093/nar/gks479 PMC3394287

[nph70728-bib-0109] Yoshimura I , Yamamoto Y , Nakano T , Finnie J . 2002. Isolation and culture of lichen photobionts and mycobionts. In: Protocols in lichenology. Berlin, Heidelberg: Springer Berlin Heidelberg, 3–33.

[nph70728-bib-0110] Yu G , Wang L‐G , Han Y , He Q‐Y . 2012. clusterProfiler: an R package for comparing biological themes among gene clusters. Omics: A Journal of Integrative Biology 16: 284–287. 22455463 10.1089/omi.2011.0118 PMC3339379

