# Supplementary material for: Chromosome‐level genome assembly of the photobiont microalga Trebouxia sp. ‘A48’ from the lichen Xanthoria parietina
Source: New Phytol. 2025 Nov 10;249(2):1036–52. doi: 10.1111/nph.70728 (PMC12712428; doi:10.1111/nph.70728)
Supplement: Supplementary file 1 — Fig. S1 Assembly of Trebouxia sp. ‘A48’ genome. Fig. S2 Metabolic map showing genes involved in carbon fixation and C4 metabolism. Fig. S3 Uncharacterized and characterized portions of the secretome in comparison to the proteome. Fig. S4 Enrichment plots showing InterProScan domains enriched in genes upregulated in: A. Lichen thalli. B. pure Trebouxia sp. ‘A48’ culture. Fig. S5 Differential gene expression between thallus parts. [file NPH-249-1036-s002.pdf]

New Phytologist

***New Phytologist* Supporting Information**

Article title: Chromosome-level genome assembly of the photobiont microalga Trebouxia sp. 'A48' from the lichen Xanthoria parietina
Authors: Gulnara Tagirdzhanova, Jasper Raistrick, and Nicholas J. Talbot
Article acceptance date: 16 October 2025

The following Supporting Information is available for this article:

Fig. S1 Assembly of Trebouxia sp. "A48" genome.

Fig. S2 Metabolic map showing genes involved in carbon fixation and C4 metabolism.

Fig. S3 Uncharacterized and characterized portions of the secretome in comparison to the proteome.

Fig. S4 Enrichment plots showing InterProScan domains enriched in genes upregulated in: A. Lichen thalli. B. pure *T.* sp. 'A48' culture.

Fig. S5 Differential gene expression between thallus parts.

Table S1 ITS sequences used for phylogenetic tree.

Table S2 Genomes used in the phylogenomic tree.

Table S3 Transcriptomic samples included in the differential gene expression analysis.

Table S4 Genes assigned to the orthogroups unique to the two genomes of Xanthoria parietina photobionts.

Table S5 Genome annotation table produced by Funannotate.

Table S6 Genes belonging to functional groups of interest: meiosis and sexual reproduction machinery, flagellum, and carbon-concentrating associated genes.

Table S7 Results of BLAST search against the NCBI nt and nr databases for the two candidate

HGT genes.

Table S8 Predicted secretome of T. sp. ‘A48’.

Table S9 Differentially expressed genes in the comparison between the algal culture and intact lichen thallus

Table S10 Output of kallisto for T. sp. 'A48' gene expression in all samples**.**

Table S11 Differentially expressed genes in the comparison between the thallus edge and center.

Fig. S1 Assembly of Trebouxia sp. “A48” genome. A. GC/coverage plot of the entire assembly. Each dot represents a contig colored based on its majority hits to the NCBI nt database. The y-axis corresponds to the coverage depth averaged across the contig, the x-axis corresponds to the GC content. The plastid genome was recovered as a single circular contig. The contigs with mitochondrial hits accounted for the majority of contigs in the assembly; these data were subsequently re-assembled to obtain a single contig assembly of the mitochondrial genome. B. Misassembly in the contig #16 visualized with IGV. The top track shows the position of the highlighted region within the contig. The track below shows the sequence of the region. The histogram shows the coverage depth for each position. The bottom track shows a portion of reads aligned to this region. Contig #16 was previously shown to have coverage than the rest of the nuclear genome and majority of hits to plastid sequences (Fig. S1a) This anomaly resulted from a misassembly, in which a nuclear contig was fused with a higher-coverage plastid contig.

C. Misassembly in the terminal region of contig #13 visualized as described above.

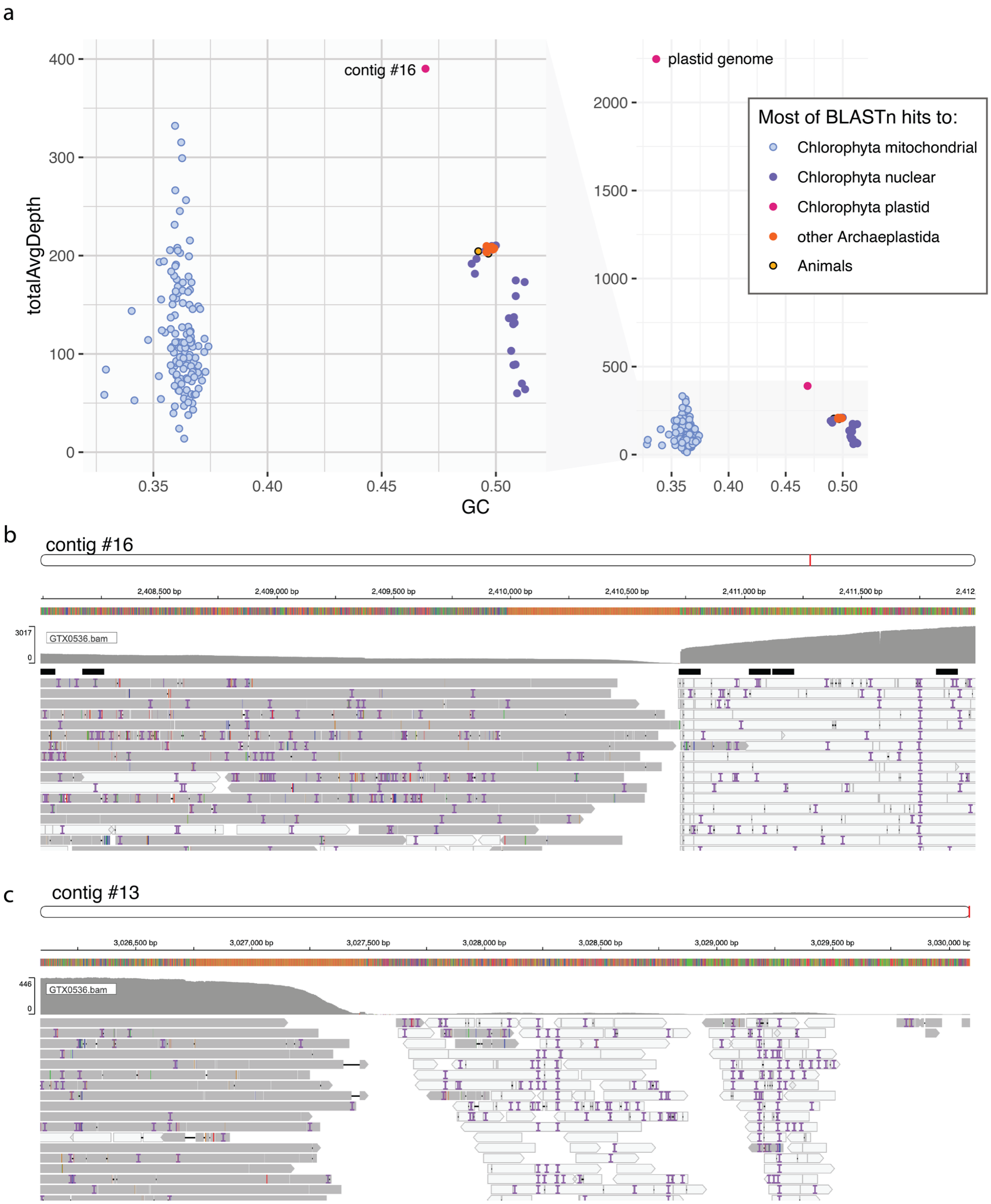

Fig. S2 Metabolic map showing genes involved in carbon fixation and C4 metabolism. Green blocks correspond to genes identified in the genome; white blocks represent absent genes. The map is generated using the KEGG Mapper Reconstruct tool (Kanehisa Laboratories).

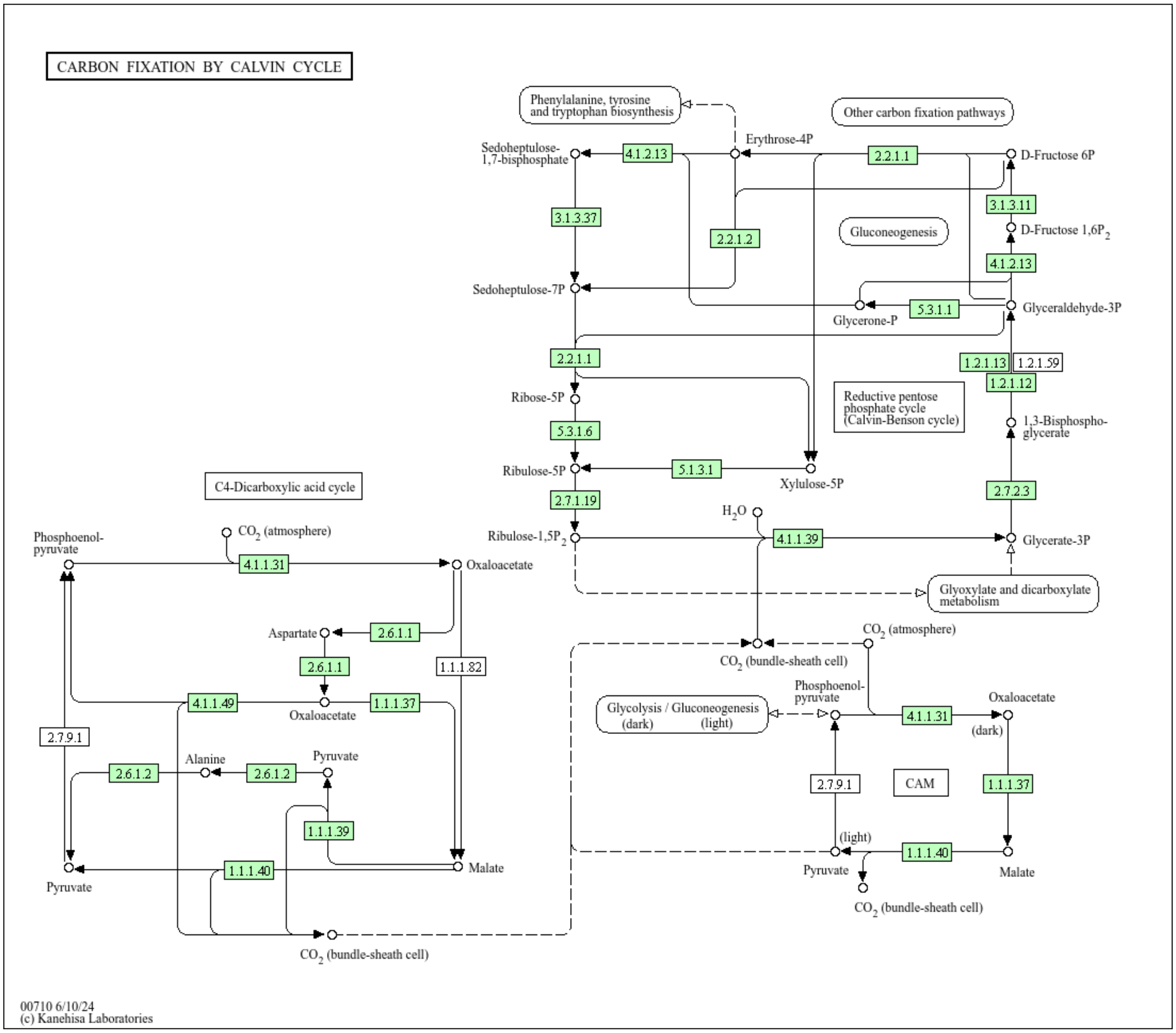

Fig. S3 Uncharacterized and characterized portions of the secretome in comparison to the proteome. A. Density plot representing length distributions. The upper panel shows the entire range; the lower panel shows distributions for proteins shorter than 1,800 amino acids. B. Distribution of cysteine content (%) across different classes of proteins.

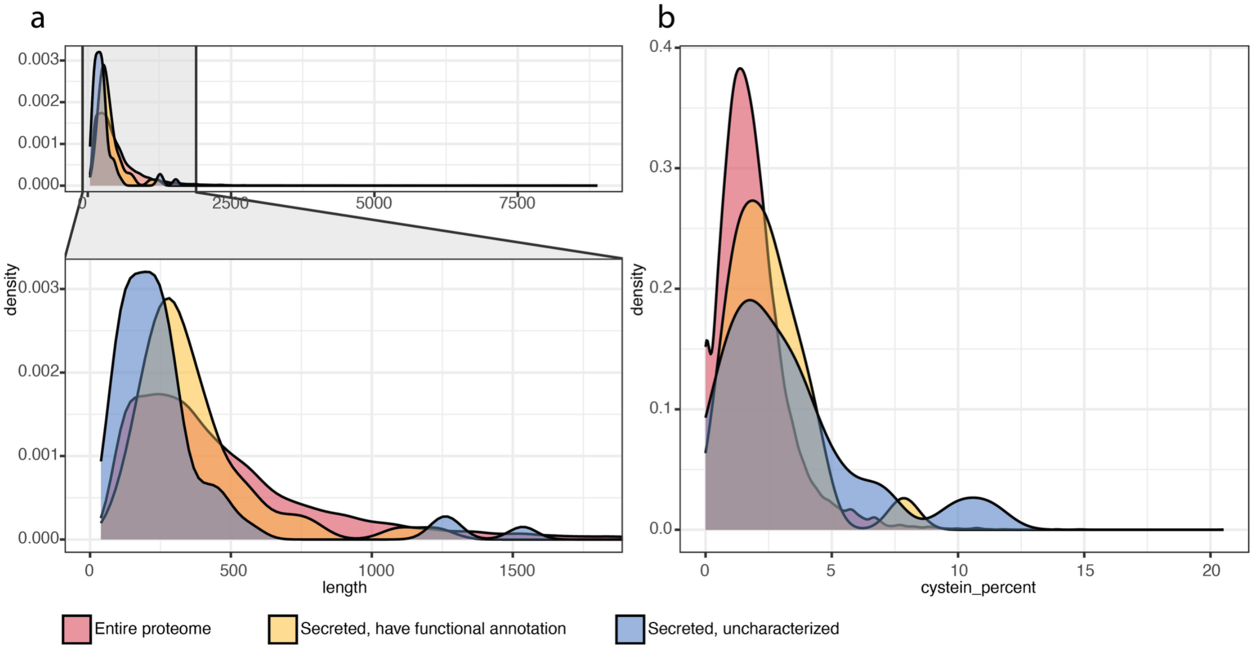

Fig. S4 Enrichment plots showing InterProScan domains enriched in genes upregulated in: A. Lichen thalli. B. pure *T.* sp. 'A48' culture. The size of the node corresponds to the number of transcripts assigned to the GO term. The color shows the enrichment score (padj-value). Two terms are linked when a single transcript is annotated with both terms.

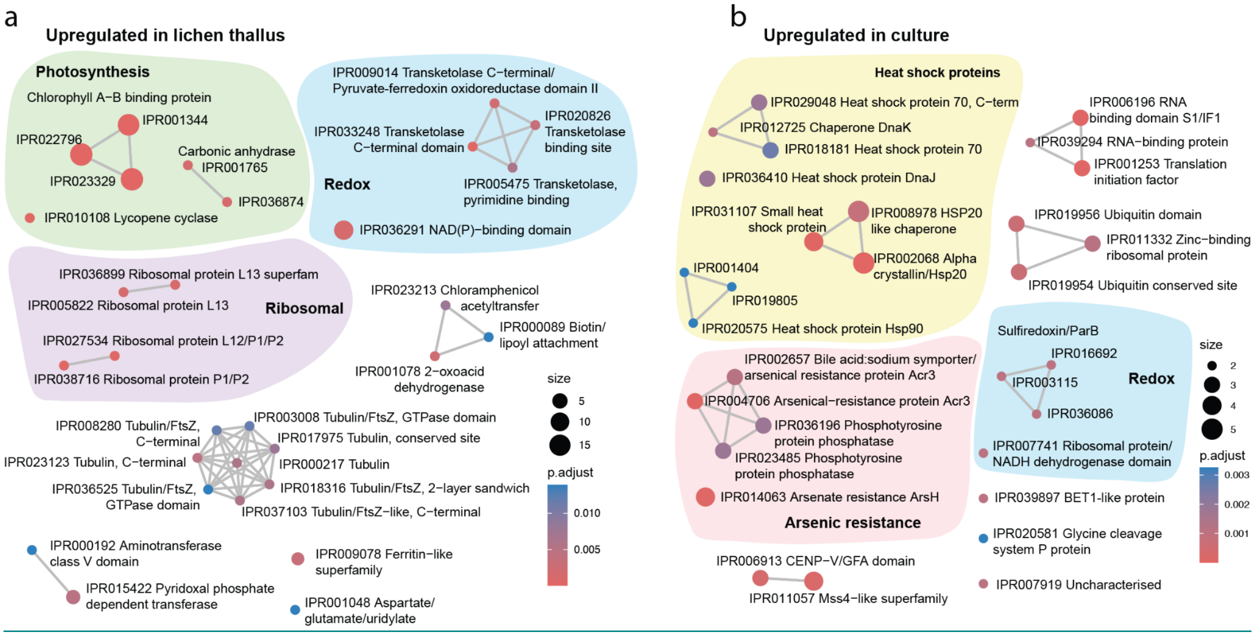

Fig. S5 Differential gene expression between thallus parts. A. Differentially-expressed genes (DEGs) and their expression levels relative to the expression levels across the transcriptome. None of the DEGs are an outlier. B. Expression of the three edge-upregulated genes annotated as chlorophyll a-b binding proteins. The samples are grouped based on the lichen thallus they derived from and colored based on the thallus part. The three shown genes were upregulated in edge compared to center. C. Sequence similarity between transcripts of the three edge-upregulated genes annotated as chlorophyll a-b binding proteins. Percent identity is calculated using the R library pwalign. D. Sequence similarity between amino acid sequences of the three edge-upregulated genes annotated as chlorophyll a-b binding proteins.

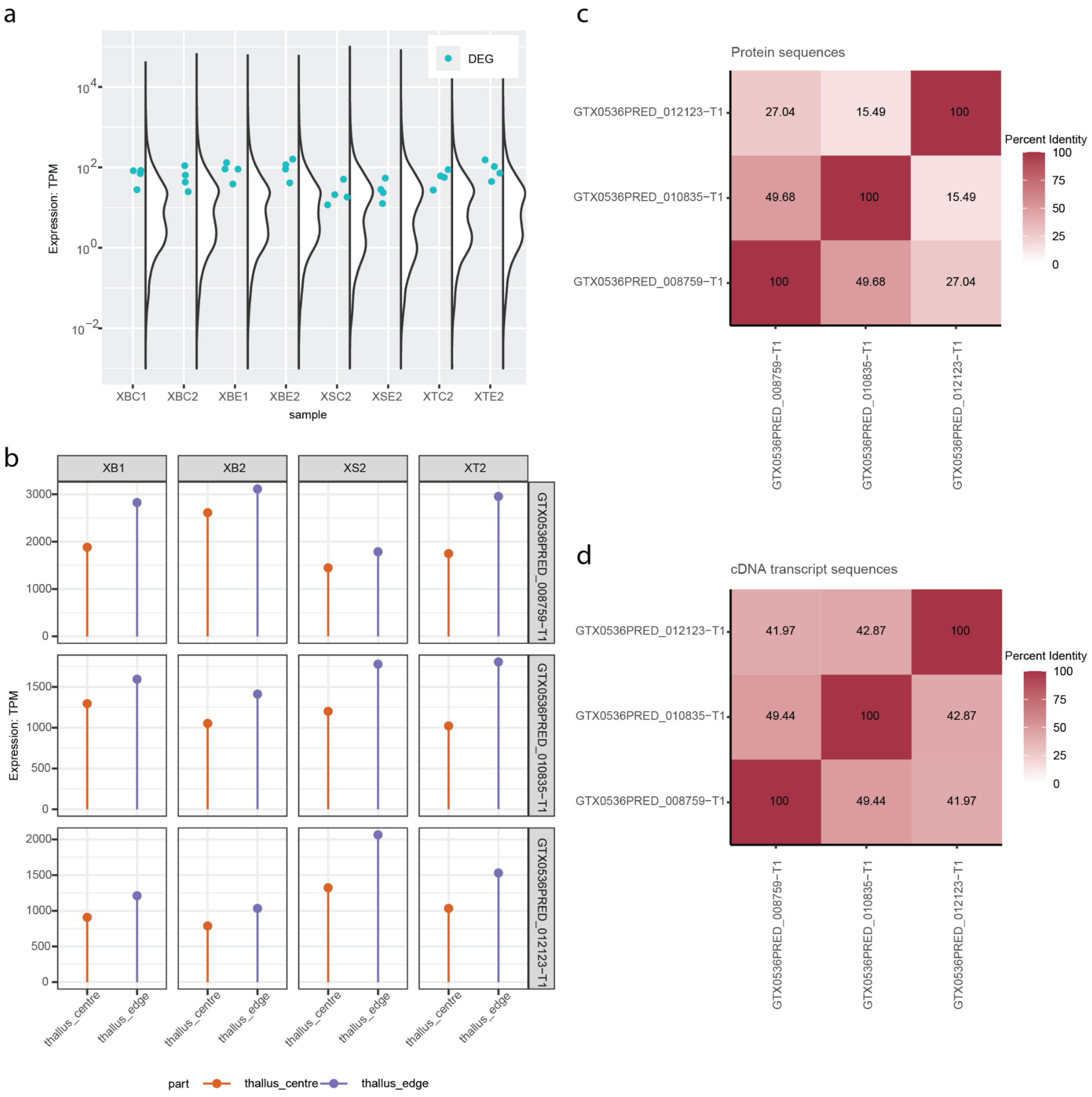

a
DEG
Expression: TPM
XBC1 XBC2 XBE1 XBE2 XSC2 XSE2 XTC2 XTE2
sample
b
XB1 XB2 XS2 XT2
GTX0536PRED_008759−T1
GTX0536PRED_010835−T1
GTX0536PRED_012123−T1
thallus_centre
thallus_edge
part
c
Protein sequences
Percent Identity
d
cDNA transcript sequences
Percent Identity

Table S1 ITS sequences used for phylogenetic tree.

Table S2 Genomes used in the phylogenomic tree.

Table S3 Transcriptomic samples included in the differential gene expression analysis.

Table S4 Genes assigned to the orthogroups unique to the two genomes of Xanthoria parietina photobionts.

Table S5 Genome annotation table produced by Funannotate.

Table S6 Genes belonging to functional groups of interest: meiosis and sexual reproduction machinery, flagellum, and carbon-concentrating associated genes.

Table S7 Results of BLAST search against the NCBI nt and nr databases for the two candidate HGT genes. Only hits with >75% query coverage are showed.

Table S8 Predicted secretome of T. sp. 'A48'. For each transcript identified as secreted, we list functional annotations based on CAZy, MEROPS (proteases), Gene Ontology, InterProScan, PFAM, and Clusters of Orthologous Genes.

Table S9 Differentially expressed genes in the comparison between the algal culture and intact lichen thallus. The table provides output from the sleuth package. B value represents the effect size (analogues to logFC); we used |b-valu|>1 threshold. For each transcript, we list functional annotations based on CAZy, MEROPS (proteases), Gene Ontology, InterProScan, PFAM, Clusters of Orthologous Genes, and secretome status.

Table S10 Output of kallisto for T. sp. 'A48' gene expression in all samples. For each target, we show both the estimated counts in any given sample, and the transcript per million number.

Table S11 Differentially expressed genes in the comparison between the thallus edge and center. The table provides output from the sleuth package. B value represents the effect size (analogues to logFC); here we provide all genes that met the p-adj<0.05 threshold, irrespective of the b-value. For each transcript, we list functional annotations based on CAZy, MEROPS (proteases), Gene Ontology, InterProScan, PFAM, Clusters of Orthologous Genes, and secretome status.
